# Use of Deep‐Learning Assisted Assessment of Cardiac Parameters in Zebrafish to Discover Cyanidin Chloride as a Novel Keap1 Inhibitor Against Doxorubicin‐Induced Cardiotoxicity

**DOI:** 10.1002/advs.202301136

**Published:** 2023-09-07

**Authors:** Changtong Liu, Yingchao Wang, Yixin Zeng, Zirong Kang, Hong Zhao, Kun Qi, Hongzhi Wu, Lu Zhao, Yi Wang

**Affiliations:** ^1^ College of Pharmaceutical Sciences Zhejiang University 866 Yuhangtang Road, Xihu District Hangzhou 310058 China; ^2^ Innovation Institute for Artificial Intelligence in Medicine of Zhejiang University 291 Fucheng Road, Qiantang District Hangzhou 310020 China; ^3^ State Key Lab of CAD&CG Zhejiang University 866 Yuhangtang Road, Xihu District Hangzhou 310058 China; ^4^ National Key Laboratory of Chinese Medicine Modernization Innovation Center of Yangtze River Delta Zhejiang University 314100 Jiaxing China

**Keywords:** cyanidin chloride, deep learning, doxorubicin, Keap1‐Nrf2 interaction, zebrafish phenotypic screen

## Abstract

Doxorubicin‐induced cardiomyopathy (DIC) brings tough clinical challenges as well as continued demand in developing agents for adjuvant cardioprotective therapies. Here, a zebrafish phenotypic screening with deep‐learning assisted multiplex cardiac functional analysis using motion videos of larval hearts is established. Through training the model on a dataset of 2125 labeled ventricular images, ZVSegNet and HRNet exhibit superior performance over previous methods. As a result of high‐content phenotypic screening, cyanidin chloride (CyCl) is identified as a potent suppressor of DIC. CyCl effectively rescues cardiac cell death and improves heart function in both in vitro and in vivo models of Doxorubicin (Dox) exposure. CyCl shows strong inhibitory effects on lipid peroxidation and mitochondrial damage and prevents ferroptosis and apoptosis‐related cell death. Molecular docking and thermal shift assay further suggest a direct binding between CyCl and Keap1, which may compete for the Keap1‐Nrf2 interaction, promote nuclear accumulation of Nrf2, and subsequentially transactivate Gpx4 and other antioxidant factors. Site‐specific mutation of R415A in Keap1 significantly attenuates the protective effects of CyCl against Dox‐induced cardiotoxicity. Taken together, the capability of deep‐learning‐assisted phenotypic screening in identifying promising lead compounds against DIC is exhibited, and new perspectives into drug discovery in the era of artificial intelligence are provided.

## Graphical abstract

The capability of deep‐learning assisted assessment of multi‐cardiac parameters in zebrafish to discover lead compounds against Doxorubicin‐induced Cardiotoxicity is demonstrated. Specifically, Cyanidin Chloride is found to potently prevent ferroptosis and apoptosis‐related cell deaths, whose effects are dependent on its strong upregulation on the antioxidative Nrf2/GPX4 pathway, which may be mediated by competitively binding to Keap1.

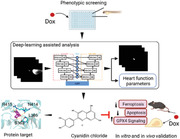

## 1 Introduction

The chemotherapeutic drug doxorubicin (Dox) showed promising therapeutic effects in a wide range of cancers including leukemia, breast cancer, and many other types of solid tumors, but its cardiac toxicity, including irreversible degenerative cardiomyopathy and congestive heart failure, severely limited its clinical application.^[^ ^1^, ^2^ ^]^ The incidence of heart failure rises with cumulative Dox doses ranging from 400–700 mg m^−2^.^[^ ^3^, ^4^ ^]^ Even at a lower dosage, some patients unavoidably suffer from heart disease many years after the initial treatment.^[^ ^5^ ^]^ Therefore, it remains of high clinical significance to clarify the underlying mechanism of Dox‐induced cardiomyopathy (DIC), as well as to develop cardioprotective therapies in Dox‐treated patients without compromising the anti‐tumor effects of Dox.

The mechanisms by which Dox elicits cardiotoxicity remain unclear. Topoisomerase‐2 (Top 2) was established as the key mediator of Dox‐induced toxic responses in cancer cells over 30 years ago, which involves the formation of DNA double‐strand breaks and apoptotic responses.^[^ ^6^, ^7^ ^]^ However, the failure in DNA damage repair could not fully explain the molecular pathogenesis of DIC. Aside from Top2‐related double‐strand breaks, excessive reactive oxygen species production (leading to damage to lipids, DNA, and proteins) and mitochondrial dysfunction have been proposed as major causes of Dox‐related cardiomyocyte damage.^[^ ^8^, ^9^ ^]^ Recently, a growing number of studies demonstrated that ferroptosis, a form of regulated cell death (RCD) characterized by iron‐dependent lipid peroxidation, is an essential mechanism of DIC.^[^ ^10^, ^11^ ^]^ Dox treatment can increase labile iron accumulation in myocardium by inactivating iron regulatory proteins and upregulating transferrin receptor.^[^ ^12^, ^13^ ^]^ In addition, Dox also downregulates the expression of the key anti‐ferroptotic protein glutathione peroxidase 4 (Gpx4), and results in excessive mitochondrial lipid peroxides.^[^ ^14^ ^]^ Dexrazoxane (DXZ), the only drug approved by U.S. Food and Drug Administration (FDA) for preventing Dox‐induced cardiotoxicity in cancer patients, is considered to work through chelating intracellular iron and blocking iron‐assisted oxidative radical production.^[^ ^15^ ^]^

In recent years, zebrafish‐based phenotypic assay is gaining increasing popularity because it more faithfully reflects the endogenous process of disease pathogenesis, compared to classical in vitro or ex vivo assays. Owing to their high fertility and low cultivation cost, zebrafish models can meet the high‐throughput needs of modern drug screening.^[^ ^16^, ^17^ ^]^ Besides, zebrafish larvae are only a few millimeters in length, which can be placed in multi‐well plates, and quickly imaged in batches through fluorescence microscopy. Nevertheless, rapid and accurate analysis of massive multimodal imaging data such as cardiac motion and hemodynamics is a key challenge for zebrafish‐based phenotypic screening. In the last few years, significant improvements have been achieved in the development of deep‐learning‐assisted automated image analysis in phenotypic screens, yet most of which were focused on cell models.^[^ ^18^, ^19^, ^20^ ^]^ There is still a lack of effective image processing tools which can automatically locate the observation target in an animal organism and perform multi‐parameter analysis. Recently, Dyballa et al reported a semi‐automatic platform that enables the rapid generation of heart rate, ejection fraction, and ventricle diameter from time‐lapse videos of zebrafish hearts. However, the users are required to manually indicate the location of a heart, and draw a line along the heart axis, from ventricle to atrium, to initiate the calculation,^[^ ^21^ ^]^ which put a limitation on the test efficiency.

In order to identify novel cardioprotective compounds against DIC, here we described a zebrafish phenotypic screening approach with deep‐learning assisted multiplex cardiac functional analysis. We proposed a pair of end‐to‐end segmentation networks (ZVSegNet and HRNet) to automatically perform heart ventricle segmentation and heart rate estimation with high accuracy and fidelity. Using this approach, we screened a medicinal herbs‐derived natural compounds library and identified cyanidin chloride (CyCl) as a potent DIC suppressor. The therapeutic benefits and mechanism of CyCl were further examined in multiple Dox‐treated cell models including H9C2 rat cardiomyocytes, primary neonatal rat cardiomyocytes (NRCMs), and human induced pluripotent stem cell‐derived cardiomyocytes (hiPSC‐CMs), as well as acute and chronic mice DIC models. This highly automated phenotypic screening strategy may accelerate the discovery of novel lead compounds in cardioprotective therapy.

## 2 Result

### 2.1 Dox‐Induced Cardiomyopathy is Primarily Mediated by Ferroptosis in Zebrafish

**Figure 1:**
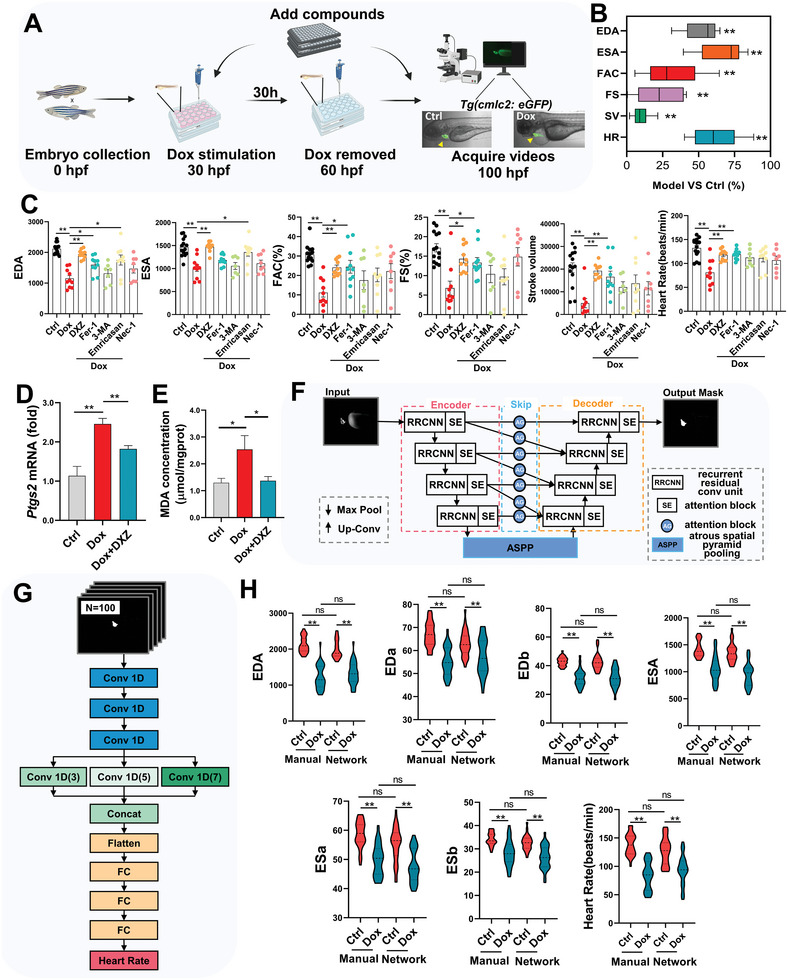
Automated cardiac function analysis in Dox‐induced cardiomyopathy zebrafish model. A) Experimental scheme of Dox stimulation and drug screening on zebrafish. Yellow arrow denotes zebrafish heart. Created with BioRender.com. B) Comparison of cardiac function between Dox‐treated group (Model) and DMSO group (Ctrl) (*n* = 10 per group). C) Quantification of zebrafish cardiac function (*n* ≥ 7 per group). D,E) Relative levels of D) *Ptgs2* and E) tissue MDA concentration, *n* = 60 to 90 per group. F–G) Network architectures of F) ZVSegNet and G) HRNet. H) Comparison of zebrafish cardiac function analysis between manual and deep‐learning assisted approaches (*n* = 30 per group). Dox, doxorubicin(65uM); DXZ, Dexrazoxane (450 µM); Fer‐1, Ferrostatin‐1(1 µM); 3‐MA, 3‐Methyladenine(250 µM); Emricasan (50 µM); Nec‐1, Necrostatin‐1(10 µM); EDA, end‐diastole area; ESA, end‐systole area; FAC, fractional area change; FS, fractional shortening; SV, stroke volume; HR, heart rate; EDa and EDb, the long axis(a) and short axis(b) of minimum‐area rectangle for EDA; ESa and ESb, the long axis(a) and short axis(b) of minimum‐area rectangle for ESA. Data are presented as the mean ± SEM. Statistical significance was analyzed using Student's t‐test in (B) and one‐way ANOVA with Tukey's post hoc test in (D,E,H); *p < 0.05, **p < 0.01, ns, non‐significant.

The zebrafish DIC model was generated as reported previously with minor modifications.^[^ ^22^ ^]^ Co‐stimulation of 65 µM Dox and 10 µM FeCl_3_ was administered to 30 hours post‐fertilization (hpf) zebrafish larvae for 30 h, and cardiac contraction videos were recorded for each larva at 100 hpf (**Figure** **1**A). A cardiomyocyte fluorescence‐labeled transgenic fish line Tg*(cmlc2: eGFP)* was used to facilitate the visualization of larval heart.^[^ ^23^ ^]^ To accurately evaluate the toxic effects of Dox on hearts, multiple cardiac functional parameters, including heart rate, end‐diastolic area (EDA), and end‐systolic area (ESA), were manually measured on the still frames of heart beating videos. Fractional area change (FAC), fractional shortening (FS), and stroke volume (SV) were calculated as described.^[^ ^24^ ^]^ After Dox exposure, the larval heart became distorted into a more elongated shape, along with severely impaired heart systolic function, decreased HR, and pericardial edema (Figure 1B, Videos S1 and S2, heartbeat videos of control (S1) and Dox‐treated(S2) larvae, Supporting Information). In addition, reduced peripheral blood flow was observed in Dox‐treated larvae, probably as a result of compromised heart pumping capability (Videos S3 and S4, blood flow videos of control(S3) and Dox‐treated (S4) larvae, Supporting Information).

Different RCD pathways have been suggested in DIC by previous studies.^[^ ^9^ ^]^ We examined what the primary cell death mechanism is that mediates Dox‐induced cardiac damage in the zebrafish model using different cell death inhibitors. Of note, DXZ and ferrostatin‐1(Fer‐1), two ferroptosis inhibitors, exhibited the most obvious rescuing effects of cardiac function, as compared with inhibitors of other RCDs, including 3‐methyladenine (an autophagy inhibitor), emricasan (a pan‐caspase inhibitor), and necrostatin‐1 (a necroptosis inhibitor) (Figure 1C). Moreover, upregulated levels of the ferroptosis biomarker prostaglandin‐endoperoxide synthase 2(Ptgs2) and the lipid peroxidation metabolite malondialdehyde (MDA) were also detected in Dox‐treated larvae, both of which can be effectively decreased by DXZ supplementation (Figure 1D,E). Therefore, these results indicated that conserved with mice models and patients, ferroptosis is a major cause of DIC in zebrafish.^[^ ^11^, ^25^ ^]^ Due to its accessibility to live heart beat imaging and feasibility of drug effects evaluation, the zebrafish larval DIC model was adopted to screen for cardioprotective compounds in the following studies.

### 2.2 ZVSegNet and HRNet Facilitated Cardiac Function Analysis on Zebrafish Heartbeat Images

Although zebrafish heart beat can be visually observed, manual inspections of cardiac function‐related parameters are tedious and unscalable. To automatedly evaluate heart function with high accuracy, we first designed a neural network ZVSegNet to accurately segment the ventricles from the heart beat videos of cardiomyocyte fluorescent‐labeled zebrafish. Heart function parameters were subsequently calculated based on the maximal and minimal ventricular sizes in a cardiac cycle. Additionally, taking as input the time‐varying ventricular area, another network HRNet is proposed to estimate the heart rate (Figure 1F,G). To train our segmentation network, we used 2125 ventricular images with manually labeled masks. The images were divided into the training/validation set with a ratio of 4:1. Data augmentation, including random rotation, flipping, and brightness/contrast adjustment (in the range of [0.9, 1.1]), is performed to further expand the training set to 5100 images. To train the heart rate estimation network, 296 zebrafish heart beating videos are used, with manually counted heart rates as the ground truth. Each video contains 100 frames and included at least 4 consecutive cardiac cycles. The dataset is also split into training/validation with a ratio of 4:1.

The segmentation network ZVSeqNet was evaluated against existing work, by computing IoU, DC, Precision, and Recall percentage on the validation set. Except for precision, which was quite close to all competing methods, our network was top‐ranked in all metrics, demonstrating the effectiveness of our architecture (Table S1, Supporting Information). The heartrate estimation network HRNet was also evaluated against existing work, via RMSE, MAE, Pearson correlation coefficient R and SD. Compared with existing approaches, our network generates results with higher accuracy and lower error standard deviation (Table S1, Supporting Information). To further evaluate the accuracy of deep learning in zebrafish cardiac function analysis, a new pool of 60 sets of randomly selected heart beat sequences (30:30 mix of control and DOX‐treated zebrafish) were analyzed manually and by deep‐learning. No significant difference was observed for all cardiac parameters between the two approaches (Figure 1H). In addition, to test the generalizability of our networks, an isoproterenol (ISO)‐treated zebrafish heart failure model was generated, with significant declines in heart rate and FS as previously reported.^[^ ^26^ ^]^ Notably, our algorithm has also demonstrated its reliability in detecting the changes of cardiac function‐related parameters in the ISO‐treated zebrafish model (15:15 mix of control and ISO‐treated zebrafish, Figure S1, Supporting Information). Taken together, our deep‐learning model is able to automatedly perform cardiac functional analysis on zebrafish heart beat images with high efficiency and accuracy.

### 2.3 Phenotypic Screening Identified Cardioprotective Natural Compounds in DIC Zebrafish

**Figure 2:**
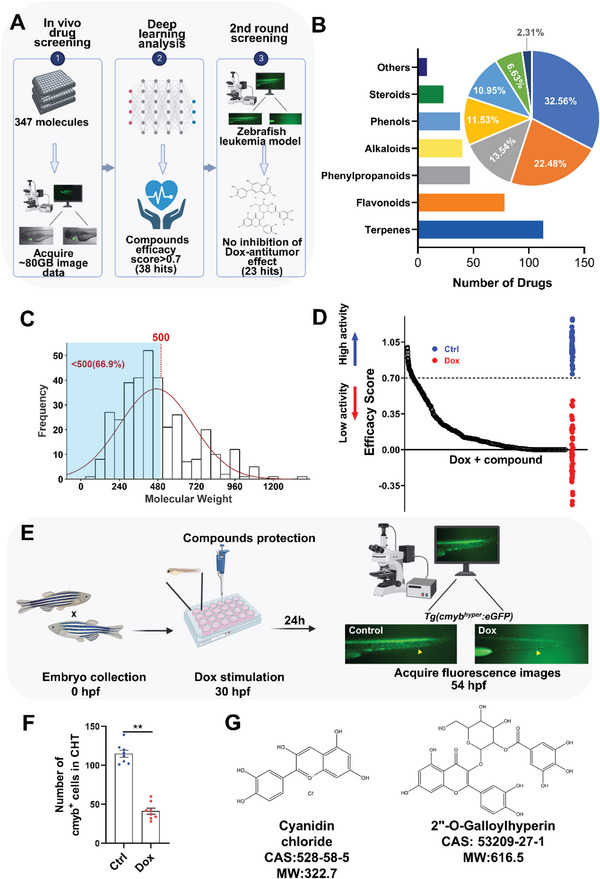
Phenotypic screening identifies the inhibitory effects of cyanidin chloride and 2“‐O‐Galloylhyperin on Dox‐induced cardiomyopathy. A) Overall scheme for the two‐round phenotypic screening. Created with BioRender.com. B) Chemical structure types and C) molecular weight distribution of natural compounds library. Those with molecular weights <500 accounted for 66.9%. D) Efficacy score of compounds against Dox‐induced cardiomyopathy in zebrafish. Dotted line shows the threshold for positive hits, which is at the level of efficacy score = 0.7(*n* ≥ 5 per compound). E) Experimental scheme for the 2nd round screen in zebrafish leukemia model. Yellow arrowhead denotes the caudal hematopoietic tissue (CHT). Created with BioRender.com. F) Quantification of the number of cmyb^+^ hematopoietic stem/progenitor cells in CHT (*n* ≥ 5 per group). G) Chemical structures of cyanidin chloride (left) and 2″‐O‐galloylhyperin (right). Quantitative data are presented as the mean ± SEM. Statistical significance was analyzed using Student's t‐test; *p < 0.05, **p < 0.01.

Natural products and their derivatives have always been an important source of lead compounds, owing to their diverse chemical structures and a wide variety of bioactivity. In order to identify novel agents that protect against DIC, we conducted an unbiased screen on zebrafish using a self‐designed medicinal herbs‐derived small molecules library including 347 natural compounds (**Figure** **2**A). The library contains 113 terpenes (32.56%), 78 flavonoids (22.48%), 47 phenylpropanoids (13.54%), 40 alkaloids (11.53%), 38 phenols (10.95%), 23 steroids (6.63%) and 8 compounds (2.31%) of other categories, with a mean molecular weight of 470.59 (Figure 2B,C). Using our deep‐learning‐based image analysis, six key cardiac parameters were automatedly obtained, including EDA, ESA, FAC, FS, SV, and heart rate. The occurrence of pericardial edema was further incorporated as an indicator of heart function, which was quickly judged by eyes and manifested as a Boolean variable (normal‐1; edema‐0). An efficacy score was calculated for each compound as described in Methods, and 38 compounds with a score higher than 0.7 were determined as positive hits (Figure 2D).

Since the aim of our study is to identify compounds capable of protecting against Dox‐induced cardiotoxicity without affecting its therapeutic effects in cancer, a second round of phenotypic screening was conducted to exclude those hits with potential influence on Dox's antitumor activity, using the *Tg*(*cmyb^hyper^:eGFP*) transgenic fish line, which is a zebrafish leukemogenesis model associated with the hyperactivation of proto‐oncogene c‐Myb (Figure 2E).^[^ ^27^ ^]^ As expected, the number of hematopoietic stem/progenitor cells decreased substantially in the caudal hematopoietic tissue (CHT) region after Dox treatment (Figure 2F). Fifteen compounds were excluded due to their significant counteractive effects on Dox's anti‐leukemia activity. Among the 23 positive hits, rosmarinic acid, CyCl, mangiferin, 2′“‐O‐galloylhyperin (2′”‐O‐GH) are the top‐ranked hits with highest efficacy scores (Table S2, Supporting Information). Notably, rosmarinic acid and mangiferin have been suggested to protect against Dox‐induced cardiac damage in murine and cell models by previous studies, supporting the high fidelity of our zebrafish DIC model in drug discovery.^[^ ^28^, ^29^ ^]^ We selected CyCl and 2″‐O‐GH for further analysis (Figure 2G).

### 2.4 Effects of Cyanidin Chloride on Dox‐induced Cardiomyocytes Toxicity In Vitro

**Figure 3:**
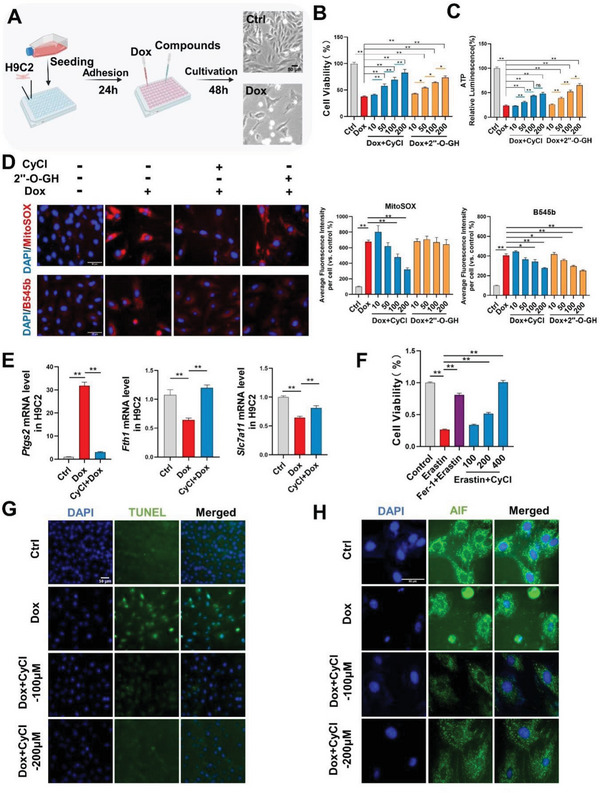
CyCl and 2″‐O‐GH rescued Dox‐induced cardiomyocyte toxicity in cultured rat cardiomyocytes (H9C2). A) Experimental scheme for Dox stimulation and compound treatment in H9C2 rat cardiomyocytes. B) Quantification of cell viability and C) ATP activity in H9C2. D) Representative fluorescence images and corresponding quantification of MitoSox and B545b in H9C2. E) Relative levels of *Ptgs2*, *Fth1*, and *Slc7a11* mRNA. The concentration of CyCl is 200 µM. F) Effect of CyCl on cell viability in Erastin‐treated H9C2. Erastin,5 µM; Fer‐1,10 µM. G–H) Representative images of TUNEL staining and AIF immunofluorescence in H9C2. Scale bar: 50 µm. Quantitative data are presented as the mean ± SEM. Statistical significance was analyzed using one‐way ANOVA with Tukey's post hoc test; *p < 0.05, **p < 0.01. ns, non‐significant.

The cardioprotective effects of CyCl and 2′“‐O‐GH were examined on H9C2 rat cardiomyocytes (**Figure** **3**A). As suggested by MTT assay and ATP measurement, Dox‐induced cell damage was significantly attenuated by both compounds in a dose‐dependent manner (Figure 3B,C). Since oxidative and nitrosative stress have been considered as main causes of DIC,^[^ ^30^, ^31^ ^]^ the intracellular stress levels of Dox‐treated cardiomyocytes were examined using a mitochondrial ROS indicator MitoSOX and an ONOO^−^ fluorogenic probe B545b^[^ ^32^ ^]^(Figure 3D). Dramatic augmentation of fluorescence intensity of both probes was detected in H9C2 cells after Dox stimulation. Treatment of either CyCl or 2′”‐O‐GH was able to relieve the increase of peroxynitrite, whereas CyCl was also able to reduce the accumulation of superoxide radicals in mitochondrial, suggesting a more general scavenging effect toward reactive species. Therefore, we focused only on CyCl for the following studies.

We next examined the effects of CyCl on regulated cell death pathways in the Dox‐treated cell model. Noticeably, the cellular expression level of *Ptgs2* was increased by over 30 folds after Dox treatment, along with a significant reduction of ferroptosis regulators ferritin heavy chain (*Fth1*) and solute carrier family 7 member 11(*Slc7a11*), suggesting the occurrence of ferroptosis. CyCl supplementation almost completely restored the normal expression of the above biomarkers (Figure 3E). The regulatory role of CyCl was further examined in the setting of erastin‐induced ferroptosis. CyCl efficiently inhibited erastin‐induced cell death in a dose‐dependent manner, demonstrating its role as a potent ferroptosis inhibitor (Figure 3F). Additionally, increased cell apoptosis was also detected in Dox‐treated H9C2 cells, as shown by elevated staining signals of DNA breakage and nuclear localization of apoptosis‐inducing factor (AIF). CyCl treatment protected cells from these pathological alterations as well (Figure 3G,H). The effects of CyCl were further evaluated on NRCMs and hiPSC‐CMs with Dox treatment (Figures S3 and S4, Supporting Information). Supportively, CyCl significantly decreased the intracellular level of reactive species and prevented cell death in both cell models. Moreover, the anti‐tumor effects of Dox in cancer cell lines including HeLa, A549, and AU565 were not influenced by CyCl (Figure S5, Supporting Information). Taken together, the above results demonstrated that CyCl can effectively rescue Dox‐induced cardiomyocyte damage in vitro without affecting its chemotherapeutic activities.

### 2.5 CyCl Protects Against DIC by Enhancing the Nrf2/Gpx4 Signaling

**Figure 4:**
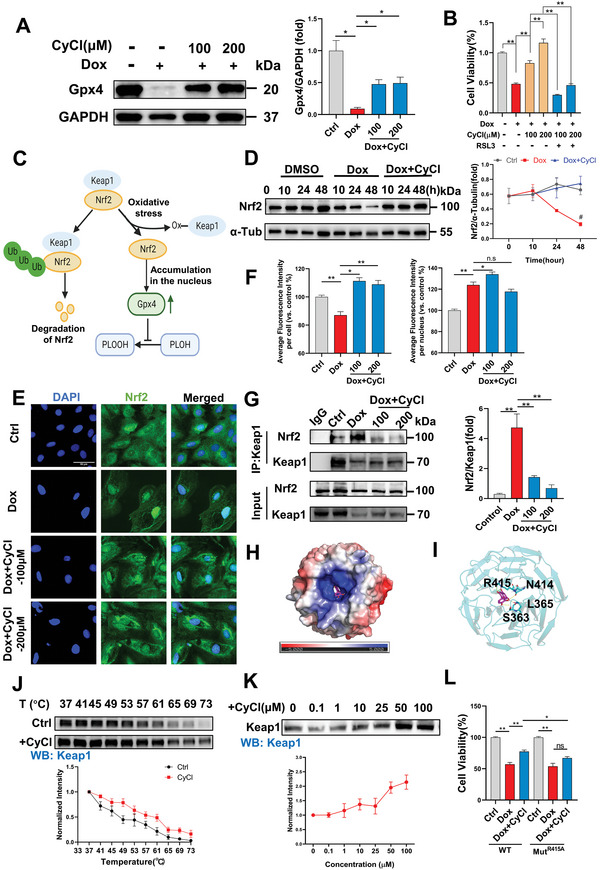
CyCl enhances Nrf2/Gpx4 signaling and directly binds to Keap1. A) Representative western blot images of GPX4 expression (*n* = 4 per group). B) Effect of RSL3 on CyCl mediated cell protection in Dox‐treated H9C2 cells. RSL3, 0.5 µM. C) Schematic model of KEAP1/NRF2/Gpx4 signaling upon oxidative stress. Created with BioRender.com. D) Representative western blot images and corresponding quantification of Nrf2 protein expression at different timepoints (*n* = 4 per group). Representative E) fluorescence images and F) corresponding quantification of Nrf2 immunofluorescence. Scar bar,50 µm. G) Co‐IP assay between KEAP1 and NRF2. H) Electrostatic potential on the surface of Keap1, with positive potential regions marked in blue and negative potential regions marked in red. I) Predicted CyCl binding sites on KEAP1. Protein‐ligand hydrogen bonds are depicted by yellow dashed lines. J) CyCl(100 µM) treatment increases the thermal stability of Keap1 in cell lysates as measured by a temperature‐dependent cellular thermal shift assay (*n* = 4 per group). K) CyCl treatment increases the thermal stability of Keap1 in cell lysates as measured by a concentration‐dependent cellular thermal shift assay at 65 °C (*n* = 4 per group). L) Effect of R415A mutation on CyCl mediated cell protection in Dox‐treated H9C2 cells. Quantitative data are presented as the mean ± SEM. Statistical significance was analyzed using a one‐way ANOVA with Tukey's post hoc test; *p < 0.05, **p < 0.01.

Gpx4 is the major enzyme catalyzing the reduction and detoxification of phospholipid hydroperoxide in mammalian cells, and thus, plays a key role in preventing ferroptotic death.^[^ ^33^ ^]^ Decline of Gpx4 was observed in cardiomyocytes following Dox treatment, whereas CyCl supplementation significantly boosted the expression of Gpx4 (**Figure** **4**A). Importantly, when the expression of Gpx4 was blocked by RAS selective lethal 3 (RSL3), a direct Gpx4 inhibitor, the rescuing effects of CyCl on Dox‐treated cardiomyocytes were almost completely abolished, suggesting that Gpx4 is required for CyCl‐mediated cardioprotection (Figure S6, Supporting Information: Figure 4B).

The endogenous expression of Gpx4 and multiple other ferroptosis regulators, such as Fth1 and Slc7a11, was targeted by the antioxidative transcription factor nuclear factor erythroid 2‐related factor 2 (Nrf2).^[^ ^34^ ^]^ Under basal conditions, Kelch‐like ECH‐associated protein 1 (Keap1) containing E3 ubiquitin ligase complex constantly targets cytoplasmic Nrf2 for proteasomal degradation. However, during increased oxidative stress, Nrf2 is allowed to accumulate in the nucleus and activate the transcription of downstream antioxidant genes (Figure 4C). Interestingly, in contrast to some previous works that reported elevated Nrf2 levels upon Dox stimulation^[^ ^35^, ^36^ ^]^ we detected decreased Nrf2 expression with prolonged Dox exposure, possibly indicating the decompensation of intracellular antioxidant machinery. Immunofluorescence staining also observed a decreased overall expression of Nrf2 in cardiomyocytes with 48 h Dox incubation. CyCl supplementation was sufficient to sustain the Nrf2 expression and promote its nuclear accumulation (Figure 4D–F). Positive regulation of CyCl on the intranuclear expression of Nrf2 was also observed in Dox‐treated hiPSC‐CMs (Figure S4, Supporting Information). In sum, the above results supported that the Nrf2/Gpx4 signaling pathway is essential for the cardioprotective effects of CyCl.

### 2.6 CyCl Competitively Binds to Keap1

Keap1‐Nrf2 protein‐protein interaction is essential for Nrf2‐mediated antioxidant responses and has become an important drug target for DIC and a number of other diseases associated with sustained oxidative damage.^[^ ^37^ ^]^ We continued to examine whether CyCl's positive regulation on Nrf2's expression is mediated through Keap1. Interestingly, no obvious change in the expression of Keap1 was observed in Dox‐treated H9C2 cells with or without CyCl treatment (Figure S7, Supporting Information). Then we analyzed the endogenous interaction between Keap1 and Nrf2 by co‐immunoprecipitation(co‐IP). Noticeably, an increased amount of Nrf2 protein was bound by Keap1 in Dox‐treated cells, whereas CyCl supplementation was able to effectively inhibit the protein interaction between Keap1 and Nrf2 (Figure 4G).

We continued to examine whether CyCl directly binds to Keap1. The Keap1‐CyCl complex was constructed by molecular docking, based on the crystal structure retrieved from the Protein Data Bank for Keap1 (PDB entry: 5FZN). The pocket of Keap1 has marked electropositive surface potential and is rich in basic arginine side chains, whereas CyCl contains acidic groups, phenolic hydroxyl, which is advantageous for Keap1 binding (Figure 4H). In particular, CyCl's acidic residues are likely to form hydrogen bonds with R415, S363, and N414, of which R415 has been reported to be an important site of Keap1‐Nrf2 protein‐protein interaction^[^ ^38^ ^]^(Figure 4I). The 2,5,7‐hydroxy‐benzopyrone group of CyCl, which extended deep inside the protein pocket, may further form a hydrogen bond with L365 (Figure 4I). Thus, CyCl may compete for Keap1's binding with the Nrf2 peptide. The direct interaction between CyCl and Keap1 was further verified by temperature‐ and dose‐dependent cellular thermal shift assays (Figure 4J,K). In addition, to examine if the binding between CyCl and Keap1 is essential for CyCl's protection against DIC, we overexpressed R415A mutated Keap1 in H9C2 cells, which is expected to disrupt the compound‐protein interaction. Notably, the rescuing effect of CyCl on Dox‐induced cardiomyocyte toxicity was significantly attenuated in cells transfected with mutated Keap1, compared with the wild‐type Keap1‐transfected group (Figure 4L). In sum, our results showed that CyCl's cardioprotective effects may be mediated through its competitive binding to Keap1.

### 2.7 CyCl Attenuates Cardiac Damage in Acute and Chronic Dox‐Treated Mice Models

**Figure 5:**
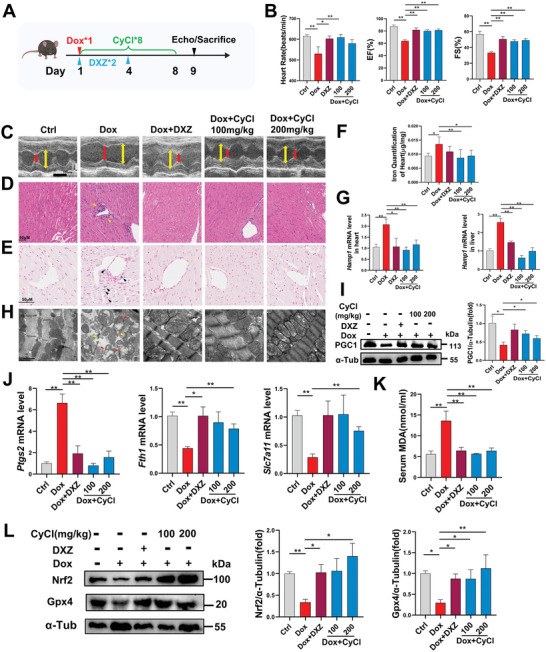
CyCl protects against cardiac damage in the acute DIC mice model. A) Experimental scheme for single dose Dox stimulation (15 mg kg^−1^) and compound treatment in mice. Created with BioRender.com. B,C) Echocardiographic analyses and representative echocardiogram images in mice. Yellow line and red line denote the left ventricular internal diameter at the end of diastole and systole respectively. Time plate is 100 ms. Scale bar is 1 mm, *n* ≥ 5 per group. EF, Ejection fraction; FS, fractional shortening. D) H&E staining and E) Prussian Blue staining of mice cardiac tissue sections. Yellow arrowhead denotes perivascular inflammatory cell infiltration. Black arrowhead denotes iron deposition. F) Iron content analysis of heart tissue. G) Transcriptional expression of *Hamp1* in the heart and liver. H) Representative transmission electron microscopy images for mice cardiac tissue. Red arrowhead denotes defective mitochondria, yellow arrowhead denotes disorganized sarcomere. I) Representative western blot of cardiac PGC1 protein expression in mice heart tissue (*n* = 6 per group). J) Relative levels of *Ptgs2*, *Fth1*, and *Slc7a11* mRNA in mice heart tissue (*n* = 3 to 4 per group). K) Serum MDA concentration in mice heart tissue (*n* = 5 to 7 per group). L) Representative western blot of cardiac Gpx4 and Nrf2 protein expression in mice hearts (*n* = 5 to 6 per group). Scale bar is 50 µm in (D,E) and 1 µm in (H). Quantitative data are presented as the mean ± SEM. Statistical significance was analyzed using a one‐way ANOVA with Tukey's post hoc test; *p < 0.05, **p < 0.01.

Finally, we examined the endogenous cardioprotective effect of CyCl in the mammalian model. Acute cardiomyopathy was induced by a single dose injection of Dox (15 mg kg^−1^) in mice, and CyCl (100 or 200 mg kg^−1^ per day) or vehicle was administered intragastrically for eight consecutive days (**Figure** **5**A). Echocardiography showed decreased heart rates, left ventricular ejection fraction (EF), and FS in the single‐dose Dox‐treated mice, which were significantly rescued by CyCl treatment, to a similar extent as those treated with DXZ (Figure 5B,C). Histological staining detected perivascular inflammatory cell infiltration in the myocardium of acute DIC mice (Figure 5D). Increased transcriptional expression of proinflammatory cytokines IL‐1β, IL‐6, and TNF‐α were also detected in the heart tissue of acute DIC mice, which may indicate the inflammatory injury related to Dox treatment (Figure S8, Supporting Information). CyCl or DXZ supplementation was able to downregulate these inflammatory responses. Besides, both Prussian blue staining and colorimetric iron quantification detected increased iron deposition in the heart tissue of acute DIC mice, which was decreased by CyCl treatment (Figure 5E,F). Moreover, the expression of *Hamp1* was increased in both the heart and liver of acute DIC mice (Figure 5G). As the encoding gene of hepcidin, the master regulator of iron homeostasis that blocks iron's intestinal absorption in response to iron overload, the elevation of *Hamp1* expression also suggested excessive iron accumulation in the two organs.^[^ ^11^ ^]^ Either CyCl or DXZ was capable to decrease *Hamp1* expression (Figure S9, Supporting Information). Furthermore, despite the largely preserved histological structure of DIC hearts, electron microscopy revealed severe ultrastructural defects, including disrupted sarcomeres and swollen mitochondria, with loss of cristae and extensive vacuolization (Figure 5H). Consistently, the expression level of peroxisome proliferator‐activated receptor γ coactivator 1 (PGC1) protein, a master regulator of mitochondrial biogenesis, was also greatly downregulated in acute DIC mice (Figure 5I). CyCl or DXZ treatment effectively alleviated these mitochondrial damages.

**Figure 6:**
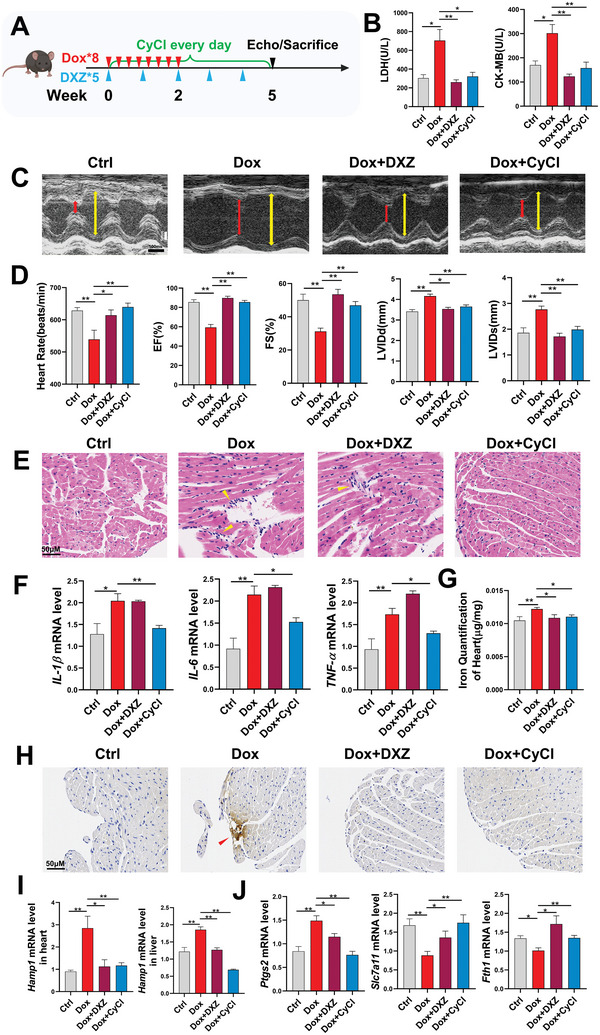
CyCl protects against cardiac damage in chronic DIC mice model. A) Experimental scheme for 2‐week Dox stimulation (3 mg kg^−1^ every other day)and compound treatment in mice. Created with BioRender.com. B) Serum levels of lactate dehydrogenase (LDH) and creatine kinase‐MB (CK‐MB). C,D) Representative echocardiogram images and echocardiographic analyses in mice. Yellow line and red line denote the left ventricular internal diameter at the end of diastole and systole respectively. Time plate is 100 ms. Scale bar is 1 mm. EF, Ejection fraction; FS, fractional shortening; LVDId and LVDIS, left ventricular internal diameter at end‐diastole and end‐systole. (*n* = 7 per group). E) H&E staining of mice cardiac tissue sections. Yellow arrowhead denotes perivascular inflammatory cell infiltration. F) QPCR levels of *IL‐1β*, *IL‐6*, and *TNF‐α* in the mice hearts. G) Iron content analysis of heart tissue. H) DAB‐enhanced Prussian Blue staining of mice cardiac tissue sections. Red arrowhead denotes iron deposition. I) Transcriptional expression of *Hamp1* in the heart and liver. J) Relative levels of *Ptgs2*, *Fth1*, and *Slc7a11* mRNA in mice heart tissue (*n* = 6–7 per group). Scale bar: 50 µm. Quantitative data are presented as the mean ± SEM. Statistical significance was analyzed using a one‐way ANOVA with Tukey's post hoc test; *p < 0.05, **p < 0.01.

Transcriptional analysis showed similar changes of ferroptosis biomarkers in acute DIC mice as those in Dox‐treated zebrafish or H9C2, including upregulation of *Ptgs2* and downregulation of *Fth1* and *Slc7a11*, supporting the major role of ferroptosis in Dox‐induced cardiotoxicity (Figure 5J). Increased expression of PTGS2 and decreased expression of SLC7A11 were detected at the protein levels (Figure S9, Supporting Information). CyCl treatment was also able to inhibit the expressional changes of ferroptosis‐related factors in the mice model. The mice's serum level of MDA was elevated after Dox stimulation and decreased by CyCl (Figure 5K). Marked reduction of Nrf2 and Gpx4 expressions were detected in the heart samples of DIC mice, which were effectively upregulated by CyCl administration (Figure 5L). In addition, we generated a chronic Dox‐treated mice model by 2‐week Dox stimulation as previously reported (**Figure** **6**A).^[^ ^39^ ^]^ No observable long‐term systemic or cardiac toxicity was observed with 5‐week CyCl treatment (Figure S10, Supporting information). By the end of the 5th week, decreased EF, FS, and increased left ventricular internal diameter at end‐systole (LVDIs) were detected by echocardiography, along with elevated levels of cardiac injury biomarkers lactate dehydrogenase (LDH) and creatine kinase‐MB (CK‐MB) in the mice serum, suggesting the development of chronic DIC. CyCl potently improved heart function and reduced myocardium injury in the chronic DIC model (Figure 6A–D). Increased inflammatory responses, local iron accumulation, and abnormal expression of ferroptosis‐related biomarkers were also suggested in chronic DIC mice, which were all significantly rescued by CyCl supplementation (Figure 6E–I). Interestingly, although DXZ is able to decrease iron overload and recover the normal expression of ferroptotic biomarkers, no obvious effect on inflammatory responses was detected for DXZ in chronic DIC mice. Taken together, these data indicated that the cardioprotective effects and regulatory mechanism of CyCl are highly conserved in mice.

## 3 Discussion

In this study, we reported an in vivo drug screening strategy that combines zebrafish live imaging with deep‐learning assisted analysis. Multifaceted phenotypic changes of heart contraction were readily observed in live zebrafish with fluorescence‐labeled cardiomyocytes, which enables the robust and reliable identification of therapeutic hits. Facilitated by neural‐network‐based ventricle segregation and heart rate estimation, we screened 347 medicinal herb‐derived natural compounds. CyCl, a bioflavonoid, was found to potently attenuate Dox‐induced cardiac cell damage in zebrafish, rat cardiomyocytes, hiPSC‐CMs, and mice DIC models, without affecting Dox's anti‐tumor activity.

Zebrafish chemical screens showed great potential in accelerating the discovery of therapeutic strategies for cardiac diseases.^[^ ^40^ ^]^ Over 96% of the genes known to cause human cardiomyopathies are conserved and highly expressed in the zebrafish heart.^[^ ^41^ ^]^ Previous studies have applied Dox to induce cardiotoxicity in zebrafish. In adult zebrafish, a single dose injection of Dox resulted in progressive cardiomyopathy starting to appear at 4 weeks after treatment, including muscular disarray, myofibril loss, and expression of stress markers, accompanied by severely reduced cardiac function and increased mortality.^[^ ^42^ ^]^ Much faster cardiotoxic responses can be observed in larvae than adults, ranging from 48–70 h as suggested by prior studies and our work,^[^ ^11^, ^22^ ^]^ which promote the usage of zebrafish in screening for cardioprotective compounds. Notably, Visnagin, a furanochromone from *Ammi visnaga*, was identified as an effective suppressor of Dox‐induced cardiotoxicity through a zebrafish‐based phenotypic screen.^[^ ^22^ ^]^ In this study, heart contraction and the blood flow of zebrafish larvae were manually categorized as “normal” or “abnormal”, and the percentage of larvae with normal appearance was used as an indicator to determine the drug efficacy. Here we combined the advantages of zebrafish in high‐throughput live imaging and deep‐learning‐assisted image analysis, to automatically extract and quantify multiple cardiac‐function‐related parameters with high accuracy and sensitivity. Our segmentation network ZVSeqNet and heartrate estimation network HRNet demonstrated superior performance over previous methods with high accuracy and efficiency.

However, it is worth mentioning that in ours and many other zebrafish‐based phenotypic screening studies, the larvae were anesthetized (most often with tricaine) to facilitate efficient imaging. Despite anesthetic treatment was equally administered to all groups and the time length of anesthesia was strictly controlled in our study to a safe range, the potential impact of anesthesia on heart function and blood circulation still cannot be completely excluded. Therefore, it is tempting to develop a high‐throughput imaging approach in unanesthetized zebrafish larvae. With the help of deep‐learning‐assisted target recognition and segmentation, such as our ZVSeqNet, the target area can be localized independently in each image frame of a video stream, and the zebrafish larva is no longer required to be still for the purpose of data analysis. However, for the imaging process by upright or inverted microscopes, the lateral‐lying position of a zebrafish larva is required to expose many anatomical regions, including the heart, and this position is much easier for a larva to maintain under anesthetized conditions. Three‐dimensional whole larva reconstruction techniques, by microscopes such as light‐sheet microscopy, may permit high‐quality imaging of any anatomical region at any body position, yet the imaging efficiency needs to be further improved for the purpose of high‐throughput screening.

CyCl is a subclass of anthocyanin that widely exists in various colored flowers, leaves, vegetables, and fruits.^[^ ^43^ ^]^ Previous studies have shown that CyCl has anti‐inflammatory and anti‐apoptosis effects, and manifested therapeutic effects in diseases including bacterial infection, osteoporosis, and diabetes.^[^ ^44^, ^45^, ^46^ ^]^ Rescuing effects of multiple anthocyanidins and anthocyanins have been observed on Dox‐treated cell models, which were related to their well‐known antioxidant properties.^[^ ^47^ ^]^ A recent study on purple corn extract also suggested beneficial role of cyanidin 3‐glucoside (C3G) on the survival of Dox‐treated mice.^[^ ^48^ ^]^ Noticeably, we also identified C3G as a positive hit in our zebrafish‐based screen, which ranked 11 with an efficacy score of 0.82 (Table S2, Supporting Information).

Multiple RCD pathways are involved in Dox‐induced cardiac damage, which contributes to the multi‐factorial mechanism of DIC and increased its biological complexity.^[^ ^9^ ^]^ Which RCD pathway has the strongest effect on Dox‐induced cardiomyocyte damage remains an open question. In our zebrafish DIC model, two ferroptosis inhibitors, DXZ and Fer‐1, outcompeted other RCD inhibitors in rescuing larval cardiac function. Consistently, superior rescuing effect of Fer‐1 was also reported in mice DIC models, as compared with agents that inhibit other cell death pathways, suggesting a fundamental role of ferroptosis in DIC pathogenesis.^[^ ^11^ ^]^ Moreover, a recent study by Liu et al. in anthracycline‐treated breast cancer patients identified hemopexin as a biomarker associated with DIC, which is a circulating glycoprotein that binds to heme and facilitates its recycling in the reticuloendothelial system.^[^ ^49^ ^]^ Hemopexin mitigates Dox‐induced ferroptosis and delivered cardiac protective function in Dox‐treated mice model. Nevertheless, increased TUNEL staining was also detected in our H9C2 model upon Dox treatment, as well as AIF's nuclear translocation, suggesting the involvement of apoptotic pathway, both caspase‐dependent and ‐independent, in Dox‐induced cardiotoxicity. CyCl was not only able to inhibit ferroptosis in Dox‐ or erastin‐treated cardiomyocytes but also rescued the above apoptotic‐related pathological changes. This can be explained by the fundamental and conserved role of lipid peroxidation in initiating different types of cell death, including both ferroptosis and apoptosis.^[^ ^50^ ^]^ For example, recent work identified a small molecule as an inducer of both ferroptosis and apoptosis, through ubiquitination of the lipid hydroperoxidase Gpx4.^[^ ^51^ ^]^

The regulatory mechanism of ferroptosis is complicated. A variety of molecular pathways were suggested in ferroptosis including redox homeostasis, iron balance, mitochondrial activity, and energy metabolism.^[^ ^33^ ^]^ Gpx4 is a major neutralizing enzyme of phospholipid hydroperoxides catalyzing lethal lipid hydroperoxides into nontoxic lipid alcohols and is identified as a key regulator in erastin‐ and RSL3‐induced ferroptosis.^[^ ^52^ ^]^ Our results suggested that Gpx4 is indispensable for CyCl‐mediated cardioprotection since the therapeutic effects of CyCl in DIC model are completely blocked by the Gpx4 inhibitor RSL3. A prior work reported significantly decreased Gpx4 in mice at day 14 following three sequential Dox administrations, as well as in cardiac cells treated with Dox for 30 h. This team further generated Gpx4 hetKO mice and found exacerbated cardiac impairments in the mice upon Dox treatment, which were believed to be mediated primarily by increased lipid peroxidation, especially in the mitochondrial membrane.^[^ ^14^ ^]^ Consistent with this, we detected downregulated protein level of Gpx4 in cell and mice models. Noticeably, severe ultrastructural defects in mitochondria were also observed in the acute DIC mice, along with decreased expression of the mitochondrial biogenesis regulator PGC1. Nrf2/Gpx4 signaling is known to protect mitochondria from oxidative injury and stimulate mitochondrial biogenesis.^[^ ^53^ ^]^ A recent study in the mice model of cardiac‐specific loss of the cytochrome c oxidase assembly factor Cox10 also linked Gpx4 deficiency and ferroptosis to oxidative phosphorylation deficiency and mitochondrial dysfunction.^[^ ^54^ ^]^ Therefore, we speculate that CyCl could protect mitochondria via its upregulation of the Nrf2/Gpx4 signaling. Nevertheless, the possibility that CyCl may also directly regulate mitochondria function through other routes cannot be excluded and warranted further study.

Gpx4 and multiple other glutathione and iron metabolism proteins are target genes of transcription factor Nrf2.^[^ ^55^ ^]^ In order to inquire how CyCl upregulates Gpx4's expression and exerts strong antioxidative effects, we examined its impact on the Keap1/Nrf2 signaling. It has been suggested by previous studies that Nrf2 signaling is adaptively activated upon Dox exposure, to protect them against oxidative cell death and confers Dox resistance in tumor cells.^[^ ^35^, ^36^ ^]^ However, we observed a progressive decline of Nrf2 expression in cardiomyocytes. CyCl is able to effectively increase Nrf2 expression in both in vivo and in vitro models. We speculate that the reduction of Nrf2 signaling may have resulted from the decompensation of endogenous anti‐oxidative system. Factors such as miR‐140‐5p were found to be significantly upregulated in rats after Dox injection, which directly targets Nrf2 and aggregate Dox‐caused myocardial oxidative damage.^[^ ^36^ ^]^ The mechanism of how cardiomyocytes make different responses at different stages of oxidative damage events still needs to be further investigated. Although it remains challenging to translate these findings on animal models to late‐onset Dox‐induced cardiomyopathy in humans, the activity of Keap1/Nrf2 signaling may have a central role in determining cell fate and clinical outcomes.

**Figure 7:**
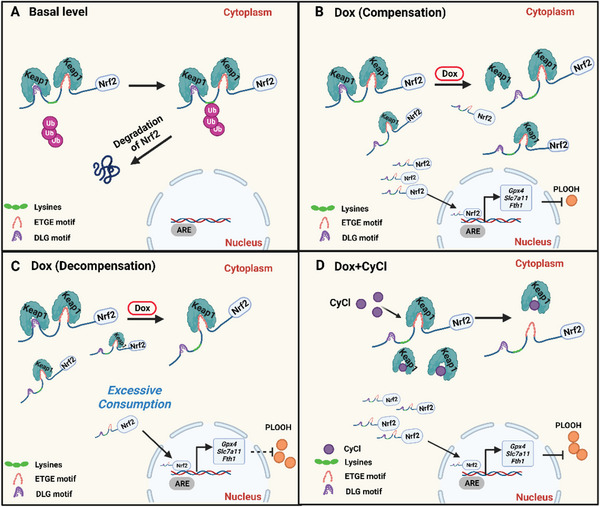
Schematic model of the proposed mechanism of CyCl's protection against Dox‐induced cardiotoxicity through competitive Keap1 binding. A) Under normal conditions, Keap1 binds to both the ETGE and DLG motifs of Nrf2. This complex configuration facilitates the ubiquitination of the lysines in the Neh2 domain of Nrf2. Nrf2 is constantly targeted for proteasomal degradation. B,C) Under Dox‐induced oxidative stress, the interaction between Keap1 and the DLG motif of Nrf2 is disrupted. Nrf2 remains trapped by Keap1 via the ETGE motif but bypasses proteasomal degradation. After the binding sites on Keap1 are occupied, newly synthesized Nrf2 is able to enter into the nucleus and bind to antioxidant response element (ARE) promoter region, to induce the transcription of its anti‐oxidative target genes, such as *Gpx4*, *Slc7a11*, and *Fth1*, which reduce the production of phospholipid hydroperoxides (PLOOH). The intracellular homeostasis can be maintained when the antioxidative machinery is sufficient to eliminate Dox‐induced oxidative stress (B, compensation), but disrupted when the level of oxidative stress exceeds the cell's scavenging capacity (C, decompensation). D) In the presence of CyCl, Nrf2 is dissociated from Keap1, as a result of the competitive binding of CyCl of Keap1. The intracellular accumulation of Nrf2 is further increased and the downstream antioxidative signaling is boosted to rescue the cell from Dox‐induced injury. Right‐handed arrow, transcriptional activation. Created with BioRender.com.

It is generally accepted that upon oxidative stress, Nrf2 is released from its binding site on Keap1 and translocates to the nucleus, where it initiates an antioxidant response. However, our co‐IP assay detected increased attachment of Nrf2 to Keap1 in Dox‐treated rat H9C2 cardiomyocytes. We think this inconsistency may be explained by the “two‐site substrate recognition model” for the Keap1‐Nrf2 interaction^[^ ^56^, ^57^ ^]^ (**Figure** **7**). According to this theory, Nrf2 interacts with Keap1 via two motifs in the Nrf2‐ECH homology 2 (Neh2) domain; the ETGE motif and the DLG motif. However, while the binding between the ETGE motif and Keap1 is strong and stable (a hinge), the binding between DLG and Keap1 is weak and less stable (a latch). This “two‐site” interaction mode is required for the exposure of the seven lysines in Neh2 domain to permit ubiquitination. Under normal conditions (Figure 7A), Nrf2 is constantly degraded and maintained at a low level. We also know, from crystallization‐based structure analysis, that ETGE and DLG motifs of Nrf2 likely interact with Keap1's Kelch domain and that the Neh2 domain of each Nrf2 molecule binds to two Keap1 molecules.^[^ ^58^, ^59^ ^]^ Upon increased cellular stress (Figure 7B), Keap1 is conformationally changed, and the DLG‐driven interaction with Nrf2 becomes disrupted, and although it remains trapped by Keap1 via ETGE, Nrf2's ubiquitination and its dynamic degradation are diminished.^[^ ^57^ ^]^ Therefore, one explanation for the increased levels of Keap1‐Nrf2 interaction demonstrated in our results (specifically in Dox‐treated H9C2 cells) is that under stress, a portion of Keap1 molecules, which are released from the Nrf2's DLG motif, may bind to other Nrf2 molecules via the high‐affinity ETGE motif. Once Keap1's binding sites become fully occupied by existing Nrf2, newly synthesized Nrf2 is then able to translocate to the nucleus, bind to antioxidant response element (ARE) promoter regions, and induce transcription of anti‐oxidative target genes. Cellular homeostasis is maintained when the intracellular antioxidative machinery can counteract Dox‐induced oxidative damage (Figure 7B), but this is disrupted when the degree of oxidative stress exceeds the cell's antioxidant capacity (Figure 7C). We therefore propose that the beneficial effects observed with CyCl, after Dox‐treatment, is the result of its competitive binding to Keap1 which releases Nrf2 which in turn translocates to the nucleus where it enhances antioxidative capacity within the cell. This leads to additional intranuclear accumulation of Nrf2 thus further protecting cells from oxidative damage (Figure 7D).

Using molecular docking and thermal shift assay, we discovered the direct binding of CyCl to Keap1, which effectively competes with Nrf2 for the major binding site on the Kelch domain, and promote the nuclear translocation of Nrf2. Hydrogen bondings are predicted between CyCl and key residues including R415, S363, and N 414, in a similar manner to the Nrf2 acidic motifs.^[^ ^60^ ^]^ Moreover, the protection of CyCl on Dox‐treated cardiomyocytes was significantly reduced when cells were overexpressed with an R415A mutated Keap1, which suggested that R415A is a key residue mediating the binding between CyCl and Keap1. Besides, by analyzing the crystal structure of binding spots mediating the interaction between previously reported small molecule inhibitors with Keap1's Kelch domain, we found that S602 is a possible hot spot. Although the predicted binding site of CyCl on Keap1 is in close proximity to S602, CyCl is less likely to have non‐covalent interaction with this residue (Figure S11A, Supporting Information).^[^ ^38^, ^61^ ^]^ Therefore, for the future structural optimization of this lead compound, it is possible to further modify the catechol group of CyCl, to strengthen its interaction with S602. Additionally, we performed a molecular docking for C3G and Keap1, and the docking score was −6.29 (Figure S11B, Supporting Information), suggesting a weaker binding compared to CyCl and Keap1(docking score: −8.02). Besides, C3G has more phenolic hydroxyl groups, which may be challenging for cell permeability.^[^ ^60^ ^]^ These may explain why CyCl has a superior cardioprotective effect than C3G.

In conclusion, our results demonstrate the strength of combining zebrafish phenotypic screening and deep‐learning analysis in discovering therapeutic candidates with cardioprotective activities from a natural compound library derived from traditional Chinese medicine herbs. With this approach, we identified CyCl as a potent suppressor of DOX‐induced cardiotoxicity and verified its effects on multiple cell and mice models. Mechanistic analysis revealed that CyCl enhanced the Nrf2/Gpx4 signaling via direct binding to Keap1. Although future studies are required to further evaluate the efficacy and metabolism of CyCl in larger mammalian models and patients, CyCl is a promising candidate for treating Dox‐induced cardiotoxicity and other forms of oxidative stress‐related heart diseases.

## 4 Conclusion

Our study proposed a pair of deep neural networks, which can automatically locate the fish ventricles and perform multiplex cardiac functional analysis. Using deep‐learning‐based phenotypic screening in zebrafish, we identified cyanidin chloride (CyCl) as a potent inhibitor of doxorubicin‐induced cardiotoxicity. Mechanistic studies revealed that CyCl potently prevents ferroptosis and apoptosis‐related cell deaths, whose effects are dependent on its strong upregulation of the antioxidative Nrf2/GPX4 pathway, which may be mediated by the competitive binding between CyCl and Nrf2's negative regulator Keap1.

## 5 Experimental Section

### Zebrafish

*Tg(cmlc2: eGFP)* and *Tg(cmyb^hyper^:eGFP)* transgenic fish was constructed by previous studies^[^ ^23^, ^62^ ^]^ and obtained from the Laboratory Animal Center of Zhejiang University. Adult male and female zebrafish were maintained under a 14 h light/10 h dark cycle at 28.5 °C with recirculating deionized water. Zebrafish were maintained following standard protocols.^[^ ^63^ ^]^ E3 medium (0.29 g L^−1^ NaCl, 0.013 g L^−1^ KCl, 0.048 g L^−1^ CaCl_2_ •2H_2_O, 0.082 g L^−1^ MgCl_2_ •6H_2_O, pH 7.2) was used as the embryo medium.

### Mice

Male C57BL/6J mice were obtained from Shanghai Slac Laboratory Animal Technology, China. All animals were fed with a standard laboratory diet and housed on a 12 h light/dark cycle at 25 °C with unrestricted access to food and water for the duration of the experiment.

All the animal experiments were performed in accordance with the Institutional Animal Care and Use Committee of Zhejiang University (Approval number: ZJU20230044) and the Guide for the Care and Use of Laboratory Animals published by the US National Institutes of Health (NIH Publication No. 85‐23, revised 1996).

### Zebrafish DIC Model

Embryos were obtained through natural spawning. *Tg(cmlc2: eGFP)* fish larvae of 30 hpf were arrayed into 24‐well plates(5 fishes per well) and were treated with Dox(65 µM; S1208, Selleck Chemicals, USA) in the presence of FeCl_3_(10 µM; 451649, Sigma‐Aldrich, USA) for 30 h. The embryo medium was then replaced with fresh E3 buffer (without Dox) and continued to culture for 40 h. At 100 hpf, fluorescent heart‐beating images were acquired by Leica DMI 3000B inversed microscope system (Leica Microsystems, Germany). For the DXZ‐treated group, 450 µM DXZ (S1222, Selleck Chemicals, USA) was supplemented in the embryo medium during and after Dox treatment from 30hpf to 100hpf. For the test with different cell death inhibitors, Fer‐1 (1 µm; HY‐100579, MedChemExpress, USA), 3‐MA (250 µM; HY‐19312, MedChemExpress, USA), Emricasan (50 µM; HY‐10396, MedChemExpress, USA), Nec‐1 (10 µM; HY‐15760, MedChemExpress, USA) were administered from 30hpf to 100 hpf.

### Zebrafish ISO Model

Embryos were obtained through natural spawning. *Tg(cmlc2: eGFP)* fish larvae were arrayed into 24‐well plates(5 fishes per well) and were treated with ISO(2 mM; HY‐B0468, MedChemExpress, USA) from 30hpf to 4dpf. At 4dpf, fluorescent heart‐beating images were acquired by Leica DMI 3000B inversed microscope system (Leica Microsystems, Germany).

### Manual Analysis of Zebrafish Cardiac Function Parameters

Zebrafish larvae were transferred into 96‐well plates with one fish per well. Each well was automatically photographed for 2 s by Leica DMI 3000B inversed microscope system (Leica Microsystems, Germany) at 50 FPS. Larvae were temporarily anesthetized in tricaine (300 µM; A5040, Sigma‐Aldrich, USA) during imaging to keep still. At least four consecutive cardiac cycles (both systolic and diastolic) were recorded. The frames of end‐systole (ES) and end‐diastole (ED) stages were manually identified as the frame after the last forward flow from the outflow tract and the frame after the last flow into the ventricle from the atrium, respectively. The ventricles were manually segmented from ES and ED frames based on the endocardium boundary indicated by cardiomyocytes fluorescence, and the number of pixels was obtained for the ventricular area of ES and ED (ESA and EDA). The ventricle area was then framed with a rotated minimum‐area rectangle and the long axis (a) and the short axis (b) were derived from the rectangle. Fractional area change (FAC), fractional shortening (FS), and stroke volume (SV) were calculated as reported before.^[^ ^24^ ^]^ The number of contraction cycles was counted based on the 2 s heart beating videos and the heart rate was calculated.

(1) $$FAC\;=\frac{EDA-ESA}{EDA}\;\times 100\%$$

(2) $$\mathrm{FS}\;=\frac{\mathrm{EDa}-\mathrm{ESa}}{\mathrm{EDa}}\;\times 100\%$$

(3) $$SV\;=\frac{4}{3}\;\times \pi \times \left(EDa\times EDb^{2}-ESa\times ESb^{2}\right)$$

(4) $$\mathrm{HR}\;=\frac{\mathrm{Number}\;\mathrm{of}\;\mathrm{Contraction}\;\mathrm{Cycles}}{\mathrm{Length}\;\mathrm{of}\;\mathrm{Video}\left(\mathrm{second}\right)}\;\times 60$$

### Ventricular Segmentation Training Database

Heart beating videos were collected by Leica DMI 3000B inversed microscope system (Leica Microsystems, Germany). For each image frame, the ventricular region was manually labeled as a binary mask, based on the endocardium boundary determined by cardiac fluorescence. A total of 2125 zebrafish ventricular image frames were labeled to construct the database.

### Heart Rates Estimation Training Database

The number of heart contractions was counted manually for each heart beating video, and the heart rates were labeled for a total of 296 video sequences.

### Segmentation Network ZVSegNet

A neural network was designed to segment zebrafish ventricles from fluorescence images. The backbone architecture was based on U‐Net.^[^ ^64^ ^]^ The original double convolution was replaced with 2‐layer recurrent residual convolutional units (RRCU)^[^ ^65^ ^]^ for more effective processing of complex features. Each skip connection was extended to two consecutive encoder levels to fuse multiple receptive fields. Encoder, decoder, and skip connections were augmented with attention blocks for focusing on learning features related to segmentation. An Atrous Spatial Pyramid Pooling (ASPP)^[^ ^66^ ^]^ layer was added for improving segmentation on samples with varying sizes. To compute ventricular axis, a rotated minimum‐area rectangle was computed to enclose the mask output from the network, and the axis lengths are derived from the rectangle.

### Heartrate Estimation Network HRNet

A network was designed to estimate heart rate from a sequence of predicted ventricular areas using our segmentation network. The heartrate network consists of 3 parts: 1) a 3‐layer CNN that extracts high‐dimensional low‐level features from the 1D input sequence; 2) an inception layer^[^ ^67^ ^]^ that transforms the low‐level features to peak features with multi‐scale information; and 3) a global analysis part (3‐layer MLP) that filters out irrelevant peaks (e.g., due to noise) and predicts the heart rate.

### Phenotype Screen on Zebrafish DIC Model

The concentration of compounds used for zebrafish DIC screen was 100 µM (at least 5 larvae for each compound) and the treatment duration was from 30hpf to 100hpf. All compounds were purchased from Shanghai Yuanye Biotechnology (China), with purities >98%. The acquired fluorescent‐labeled zebrafish heartbeat videos were analyzed by the deep‐learning‐based network to compute the cardiac function parameters. The occurrence of pericardial edema was judged by eyes and manifested as a Boolean variable (normal‐1; edema‐0). The anti‐DIC activity of each compound was evaluated by the efficacy score.

(5) $$\mathrm{Rescue}\;\mathrm{Index}\;=\frac{X_{\mathrm{drug}}-X_{\mathrm{model}}}{X_{\mathrm{ctrl}}-X_{\mathrm{model}}}$$

(6) $$\mathrm{Efficacy}\;\mathrm{Score}\;=\frac{\sum \mathrm{Rescue}\;\mathrm{Index}}{\mathrm{Number}\;\mathrm{of}\;\mathrm{parameters}}$$

### Phenotype Screen on Zebrafish Leukemia Model

Larvae were obtained through natural spawning. *Tg(cmyb^hyper^:eGFP*) fish larvae of 30 h‐post fertilization (hpf) were arrayed into 24‐well plates(5 fishes per well) and were treated with 0.1% DMSO (control), Dox(65 µM), or compounds(100 µM) for 24 h (*n*≥ 5 each group). The embryos were imaged at 54hpf, and the number of *cmyb* ^+^ cells in the aorta‐gonad‐mesonephros hematopoiesis region was counted manually.

### Cell Culture and Treatment

The rat cardiomyocyte cell line H9C2 was obtained from Nanjing Beretti Biological Technology (China), and cultured in high glucose DMEM with 10% FBS and antibiotics (100 units mL^−1^ penicillin and 100 µg mL^−1^ streptomycin) in a humidified atmosphere at 37 °C and 5% CO_2_. Primary cultures of NRCMs were prepared from the hearts of neonatal rats (1 to 3 days old, Sprague‐Dawley rats, either sex) using the Neonatal Heart Dissociation Kit (130‐098‐373, MACS, Germany) according to the manufacturer's protocol. Cardiomyocyte cultures were plated in gelatin‐coated plates in a plating medium containing 10% FBS. Cells were pre‐plated for 0.5 h to remove fibroblasts, and unattached cardiomyocytes in suspension were collected and plated in fibronectin‐coated culture plates. Cardiomyocyte cultures were used after 24 h of plating. Frozen HiPSC‐CMs were purchased from Ronovation Biotech (RCCM‐LINGYIN‐011, China), and cultured in RPMI 1640 and B‐27 supplement with insulin (11875093+17504044, Gibco, USA) for three days before drug treatment.

For Dox treatment, H9C2, NRCMs, or HiPSC‐CMs were seeded into 96‐well plates in 3000, 8000, or 10 000 cells per well, respectively. Dox (0.5 µM) and FeCl_3_(0.1 µM) were added for 48 h after cells adhered to the wells. DMSO (0.1%) was added as the blank control. For erastin or RSL3 treatment, H9C2 was treated with erastin (5 µM; HY‐15763, MedChemExpress, USA) or RSL3(0.5 µM; HY‐100218A, MedChemExpress, USA) for 48 h. For rescue experiments in vitro, compounds were used at the doses indicated in each figure.

### Cell Viability Assay and ATP Measurement

For the cell viability assay, the medium was removed and the cells were incubated with MTT solution (0.5 mg mL^−1^; M2128, Sigma‐Aldrich, USA) for 4 h at 37 °C. 100 µl DMSO was added to each well and incubated for 10 min at 37 °C with vibration at 300 rpm. The absorbance was measured at 570 nm using Tecan Infinite M1000 PRO microplate reader (Tecan, Switzerland).

ATP level was determined using the CellTiter‐Glo luminescent Cell Viability Assay (G7570, Promega, USA,) according to the manufacturer's instructions. After treatment, the luminescence was recorded using Tecan Infinite M1000 PRO microplate reader.

### Measurements of Intracellular Nitrosative Stress and Mitochondrial ROS

H9C2 cells were plated in 96‐well plates and treated with the abovementioned method. B545b probe was a gift from Prof. Xin Li,^[^ ^32^ ^]^ and MitoSOX Red Mitochondrial Superoxide Indicator was purchased from Yeasen Biotech(40778ES50, China). The cells were stained according to the instructions and were imaged with the ImageXpress Pico Automated Cell Imaging System (Molecular Devices, USA).

### TUNEL Staining

Cells were fixed in 4% paraformaldehyde for 30 min, rinsed with PBST three times, and incubated with 0.3% Triton X‐100 in PBS for 5 min at room temperature. Cells were then stained with Colorimetric TUNEL Apoptosis Assay Kit (C1086, Beyotime, China). Fluorescent images were acquired using MetaXpress High‐Content Image (Molecular Devices, USA).

### Immunofluorescence Staining

Cells were fixed with 4% paraformaldehyde for 30 min at room temperature, permeabilized with 0.1% Triton X‐100 for 5 min, and blocked with 5% BSA for 1 h. Anti‐AIF (67791‐1‐Ig, Proteintech, USA) or anti‐Nrf2 (AF7623, Beyotime, China) antibodies were used at 1:200 and incubated overnight at 4 °C. Then the cells were stained with FITC‐labeled Goat Anti‐Rabbit IgG (H+L) (1:500) for 60 min at room temperature. Fluorescent images were acquired with Leica DMI 3000B inversed microscope system (Leica Microsystems, Germany). Image quantitative analysis was performed with Image J software (2.0.0). The average cellular intensity was quantified by dividing the total fluorescence intensity by the number of cells. The average nuclear intensity was quantified by dividing the total fluorescence intensity of the FITC channel in the nucleus by the number of cells.

### Quantitative RT‐PCR

Total RNA was isolated from zebrafish larvae, H9C2 cells, or tissue using an RNA‐Quick Purification Kit (RN001, ES Science, China), and then converted to single‐strand cDNA with HiFiScript cDNA Synthesis Kit (CW2569M, CWBIO, China). Real‐time PCR was performed using the two‐step quantitative RT‐PCR method with 2 X SYBR Green qPCR Mater Mix (B21202, Bimake, USA). The sequences of all primers used in the study were listed in Table S3 (Supporting Information).

### MDA Analysis

Zebrafish larvae or mice heart tissue were collected and grinded manually in PBS. The level of MDA was analyzed by the MDA Detection Kit (S0131, Beyotime, China), and measured by Tecan Infinite M1000 PRO microplate reader. At least 20–30 zebrafish larvae were measured as one biological repeat and 3 biological repeats were performed for each treatment condition.

### Western Blot

Myocardial tissue (≈20 mg) was lysed in cold RIPA lysis buffer containing 1% phenylmethylsulfonyl fluoride (PMSF; ST505, Beyotime, China) and 1% protease inhibitor cocktail (HY‐K0010, MedChemExpress, USA) to extract the whole protein. H9C2 was lysed in cold IP lysis buffer with 1% PMSF to extract the whole protein. The lysate was centrifuged at 12 000 g for 10 min at 4 °C and the protein concentration was determined by BCA Protein Assay Kit (23227, Thermo Fisher, USA). After washing with cold PBS, equal amounts of protein samples were separated on SDS‐PAGE gels and transferred to a polyvinylidene difluoride membrane. Then, the membrane was incubated with primary antibodies against Nrf2(AF7623, Beyotime, China, 1:1000), α‐Tubulin (AF0001, Beyotime, China, 1:1000), GAPDH (AG019, Beyotime, China, 1:1000), Keap1(60027‐1‐Ig, Proteintech, USA, 1:1000), GPX4 (67763‐1‐Ig, Proteintech, USA, 1:1000), PGC‐1(ab188102, Abcam, U.K., 1:1000), PTGS2 (66351‐1‐Ig, Proteintech, USA), or SLC7A11 (AF7992, Beyotime, China) overnight at 4 °C after been blocked in TBST containing 5% skim milk. After incubated with secondary antibodies (Diluted in 5% BSA at 1:5000) for 1 h at room temperature, the membranes were treated with ECL chemiluminescence reagents. The blot intensity was quantified with a ChemiDoc MP Imaging System (12003154, Bio‐Rad, U.S.A).

### Co‐Immunoprecipitation (co‐IP) Assay

H9C2 cells were lysed in cold IP lysis buffer containing 1% PMSF. The lysates were then incubated with indicated anti‐Keap1 or anti‐IgG antibody (1:200) at 4 °C overnight. 30 µL of protein A/G beads (HY‐K0202, MedChemExpress, USA) were added for another 2 h on a rotator. Immunoprecipitants were centrifuged and washed six times with wash buffer at 4 °C.

### Temperature‐Dependent Thermal Shift Assay

H9C2 lysate was incubated with 100 µM CyCl at 4 °C for 4 h and divided into ten groups of 50 µL each. The samples were incubated at a temperature gradient of 37, 41, 45, 49, 53, 57, 61, 65, 69, and 73 °C for 4 min, cooled at room temperature for 3 min, then centrifuged at 12 000 g for 10 min at 4 °C. The supernatant was collected and analyzed by western blotting.

### Concentration‐Dependent Protein Thermal Shift Assay

H9C2 lysate was divided equally into 7 samples and was incubated with different concentrations of CyCl (0, 0.1, 1, 10, 25, 50, 100 µM) at 4 °C for 4 h. Each group of samples was incubated at 65 °C for 4 min, cooled at room temperature for 3 min, and then centrifuged at 12 000 g for 10 min at 4 °C. The supernatant was collected and analyzed by western blotting.

### Docking

The initial crystal structure of Keap1 (PDB: 5FZN) was obtained from Protein Data Bank, and prepared by the Protein Preparation Wizard in Schrödinger 2018 to ensure that the root‐mean‐square deviation of the protein reached a maximum value of 0.3 Å. The molecular structure of CyCl was preprocessed by the Ligprep module. Next, the scoring grid for docking was generated by enclosing the residues in a box with a size of 15 Å × 15 Å × 15 Å centered on the original ligand using the Receptor Grid Generation module with default settings.

The Glide module with Extra precision (XP) scoring mode was used to predict the binding poses of the prepared small molecules. The confirmation with the highest score was selected for further analysis.

### Acute Mouse DIC Model

Eight weeks old mice were intraperitoneally injected with Dox (15 mg kg^−1^) to generate the DIC model. The Dox+ DXZ group mice were intraperitoneally injected with 60 mg kg^−1^ DXZ on the first and fourth days. Mice in the Dox+CyCl group were intragastrically administered with 100 or 200 mg kg^−1^ d^−1^ CyCl (dissolved in 0.5%CMC‐Na) for 8 days. Echocardiography was performed using a Vevo 2100 system (VisualSonics, USA) on the 9th day after Dox injection and the mice were sacrificed afterward for further analysis.

### Chronic Mouse DIC Model

Eight weeks old mice were intraperitoneally injected with Dox (3 mg kg^−1^) twice a day for 2 weeks to generate the chronic DIC model. The Dox+DXZ group mice were intraperitoneally injected with 60 mg kg^−1^ DXZ once a week. Mice in the Dox+CyCl group were intragastrically administered with 50 mg kg^−1^ CyCl (dissolved in 0.5%CMC‐Na) every day. Echocardiography was performed using a Vevo 2100 system (VisualSonics, USA) by the end of the 5th week after the initiation of Dox stimulation and the mice were sacrificed afterward for further analysis.

### Transmission Electron Microscopy

Briefly, the tissue was first fixed with 2.5% glutaraldehyde in 0.1 m phosphate buffer for >4 h, washed three times in the phosphate buffer, subsequently fixed with 1% OsO4 in phosphate buffer, dehydrated in a gradient series of acetone, and infiltrated and embedded using Spurr low‐viscosity embedding kit (EM0300, Sigma‐Aldrich, USA). Finally, the samples were cut into thin sections of 70 nm thickness and stained with uranyl acetate and lead citrate. Images were captured with Tecnai G2 spirit 120 kV TEM (Thermo FEI, USA).

### Histological Analysis

Hearts were fixed overnight in 4% paraformaldehyde (pH 7.4), embedded in paraffin, and serially sectioned at 5‐µm thickness. The sections were stained with Hematoxylin and Eosin (H&E) to assess morphological changes. The iron content was assayed by regular Prussian blue staining in the acute mice DIC model; and by DAB‐enhanced Prussian blue staining in the chronic DIC model.

### Colorimetric Iron Quantification Analysis

The mice heart tissue was weighted and homogenized on an ice bath. The supernatant was tested using the tissue iron content assay kit (BC4355, Solarbio, China) according to the manufacturer's protocol.

### Statistical Analysis

The data were interpreted as means ± SEM of three or more independent experiments. Statistical analyses were performed with an unpaired two‐tailed *t*‐test for comparisons between two groups, one‐way analysis of variance (ANOVA), and Turkey's post hoc test for comparisons among more than two groups. All analyses were carried out using GraphPad Prism 8.0.2(USA). *p*‐values <0.05 were considered statistically significant.

## Conflict of Interest

The authors declare no conflict of interest.

## Supporting information

Supporting Information

Supporting Information

Supporting Information

Supporting Information

Supporting Information

## Data Availability

The data that support the findings of this study are available from the corresponding author upon reasonable request.
