# Supplementary material for: Use of Deep‐Learning Assisted Assessment of Cardiac Parameters in Zebrafish to Discover Cyanidin Chloride as a Novel Keap1 Inhibitor Against Doxorubicin‐Induced Cardiotoxicity
Source: Adv Sci (Weinh). 2023 Sep 7;10(30):2301136. doi: 10.1002/advs.202301136 (PMC10602559; doi:10.1002/advs.202301136)
Supplement: Supplementary file 1 — Supporting Information [file ADVS-10-2301136-s002.pdf]

ADVANCED SCIENCE
Open Access

Supporting Information

for *Adv. Sci.*, DOI 10.1002/advs.202301136

Use of Deep-Learning Assisted Assessment of Cardiac Parameters in Zebrafish to Discover Cyanidin Chloride as a Novel Keap1 Inhibitor Against Doxorubicin-Induced Cardiotoxicity

Changtong Liu, Yingchao Wang, Yixin Zeng, Zirong Kang, Hong Zhao, Kun Qi, Hongzhi Wu, Lu Zhao* and Yi Wang**

Supporting Information

Deep-learning Assisted Assessment of Cardiac Parameters in Zebrafish to Discovering Cyanidin Chloride as a Novel Keap1 Inhibitor against Doxorubicin-induced Cardiotoxicity

Changtong Liu[1], Yingchao Wang[1,2], Yixin Zeng[3], Zirong Kang[3], Hong Zhao[1], Kun Qi[1], Hongzhi Wu[3]*, Lu Zhao[1]*, Yi Wang[1,2,4]*

[1] College of Pharmaceutical Sciences, Zhejiang University, 866 Yuhangtang Road, Xihu District, Hangzhou 310058, China
[2] Innovation Institute for Artificial Intelligence in Medicine of Zhejiang University, 291 Fucheng Road, Qiantang District, Hangzhou 310020, China
[3] State Key Lab of CAD&CG, Zhejiang University, 866 Yuhangtang Road, Xihu District, Hangzhou 310058, China
[4] National Key Laboratory of Chinese Medicine Modernization, Innovation Center of Yangtze River Delta, Zhejiang University, 314100, Jiaxing, China.

*Corresponding author. E-mail addresses: hwu@zju.edu.cn (H.Wu), lzhao@zju.edu.cn (L.Zhao), zjuwangyi@zju.edu.cn (Y.Wang).

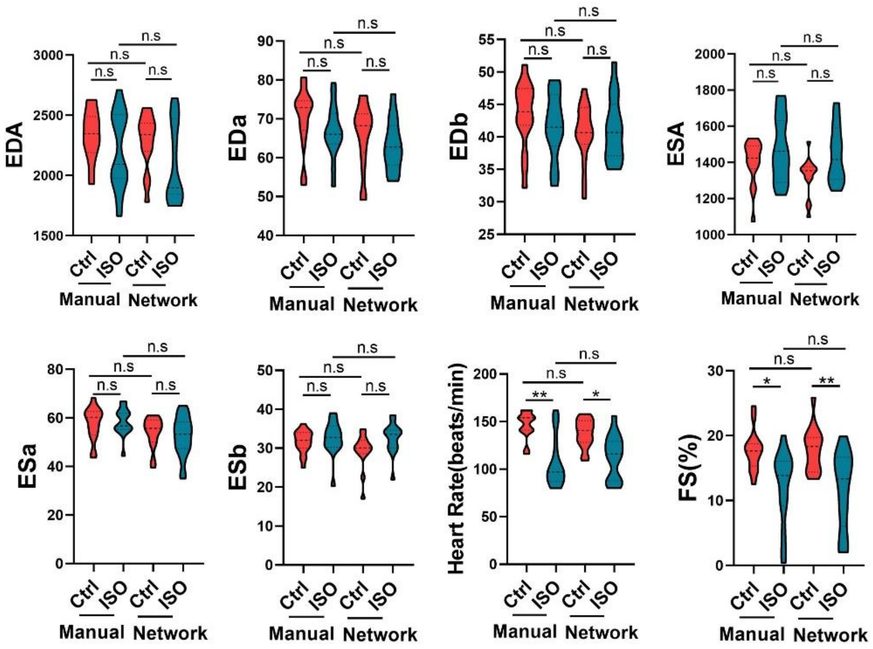

Supporting Information Figure S1. Comparison of zebrafish cardiac function analysis between manual and deep-learning assisted approaches (n=15) in the isoproterenol (ISO)-treated zebrafish model. EDA, end-diastole area; ESA, end-systole area; EDa and EDb, the long axis(a) and short axis(b) of minimum-area rectangle for EDA; ESa and ESb, the long axis(a) and short axis(b) of minimum-area rectangle for ESA; FS, fractional shortening; HR, heart rate. Statistical significance was analyzed using one-way ANOVA with Tukey's post hoc test; *P < 0.05, **P < 0.01, ns, non-significant.

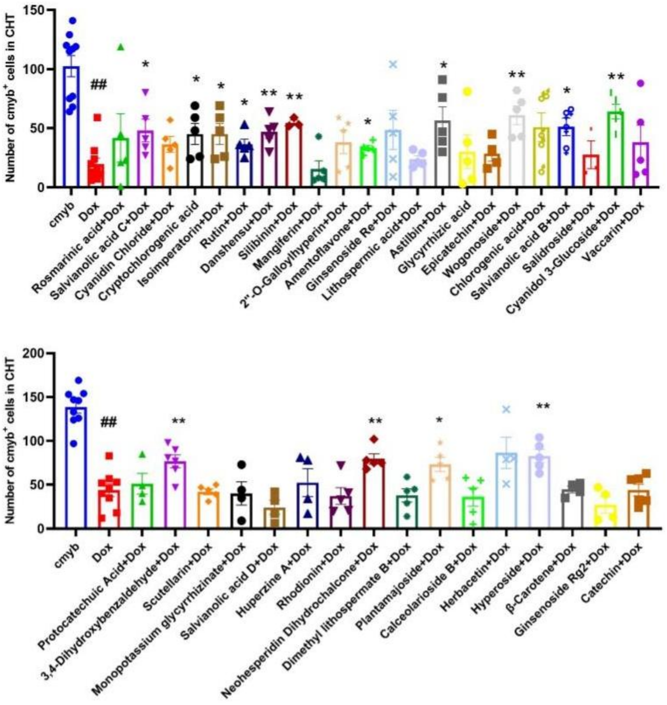

Supporting Information Figure S2. Phenotypic screen in zebrafish leukemia model. Effects of compounds on the anti-leukemia activity of Dox in zebrafish. Quantitative data are presented as the mean ± SEM (n ≥ 5 per group). Statistical significance was analyzed using one-way ANOVA with Tukey's post hoc test; #, compared with the control group, *, compared with the Dox-treated model group. *P < 0.05, **or ##P < 0.01.

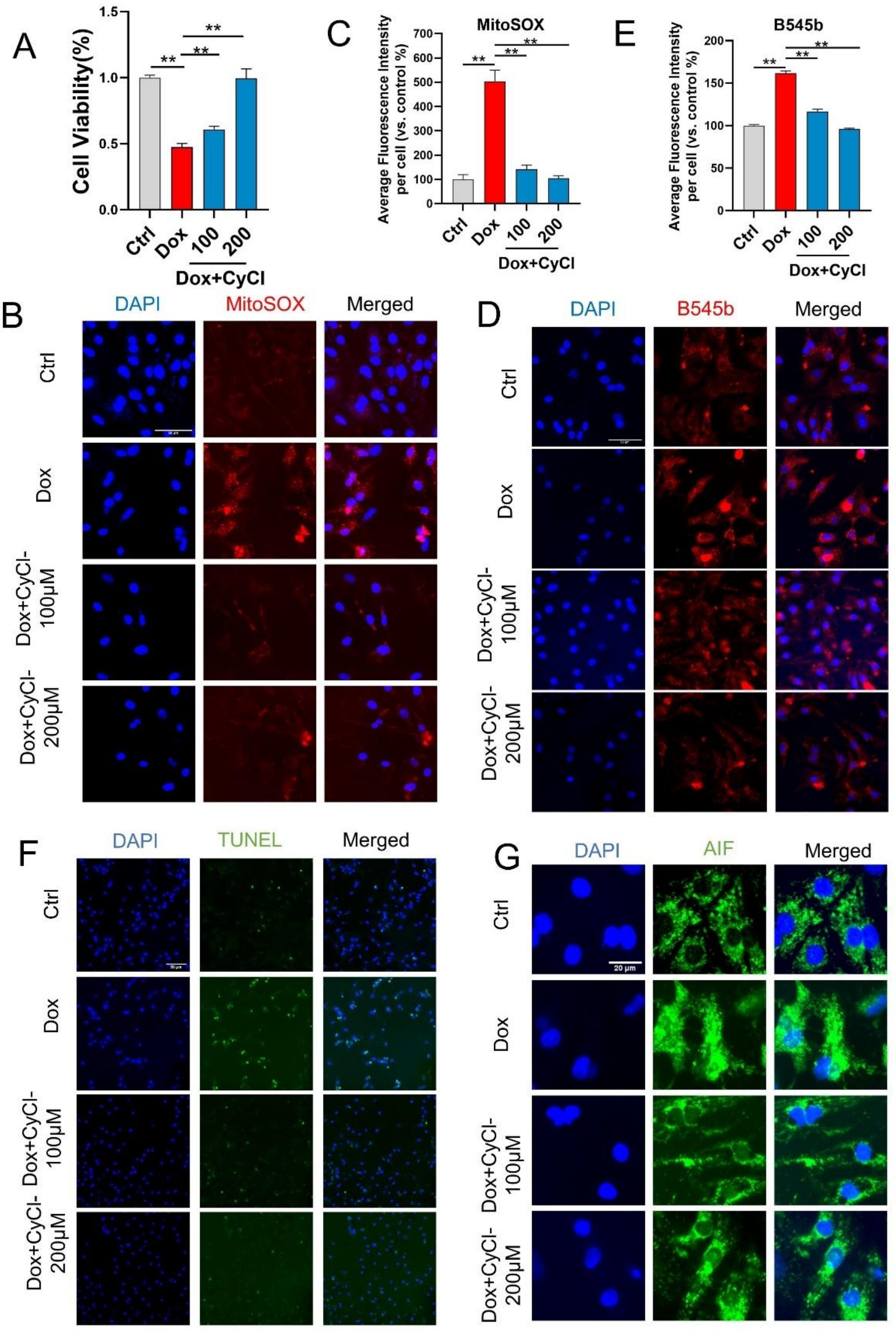

Supporting Information Figure S3. CyCl rescued Dox-induced cardiomyocytes injury in primary neonatal rat cardiomyocytes (NRCMs). (A) Quantification of cell viability in NRCMs. (B-E) Representative fluorescence images and corresponding quantification of MitoSox and B545b in NRCMs. (F-G) Representative images of TUNEL staining and of AIF immunofluorescence in NRCMs. Scale bar: 50μm in (B,D,F) and 20μm in (G). Quantitative data are presented as the mean ± SEM. Statistical significance was analyzed using one-way ANOVA with Tukey's post hoc test; **P < 0.01.

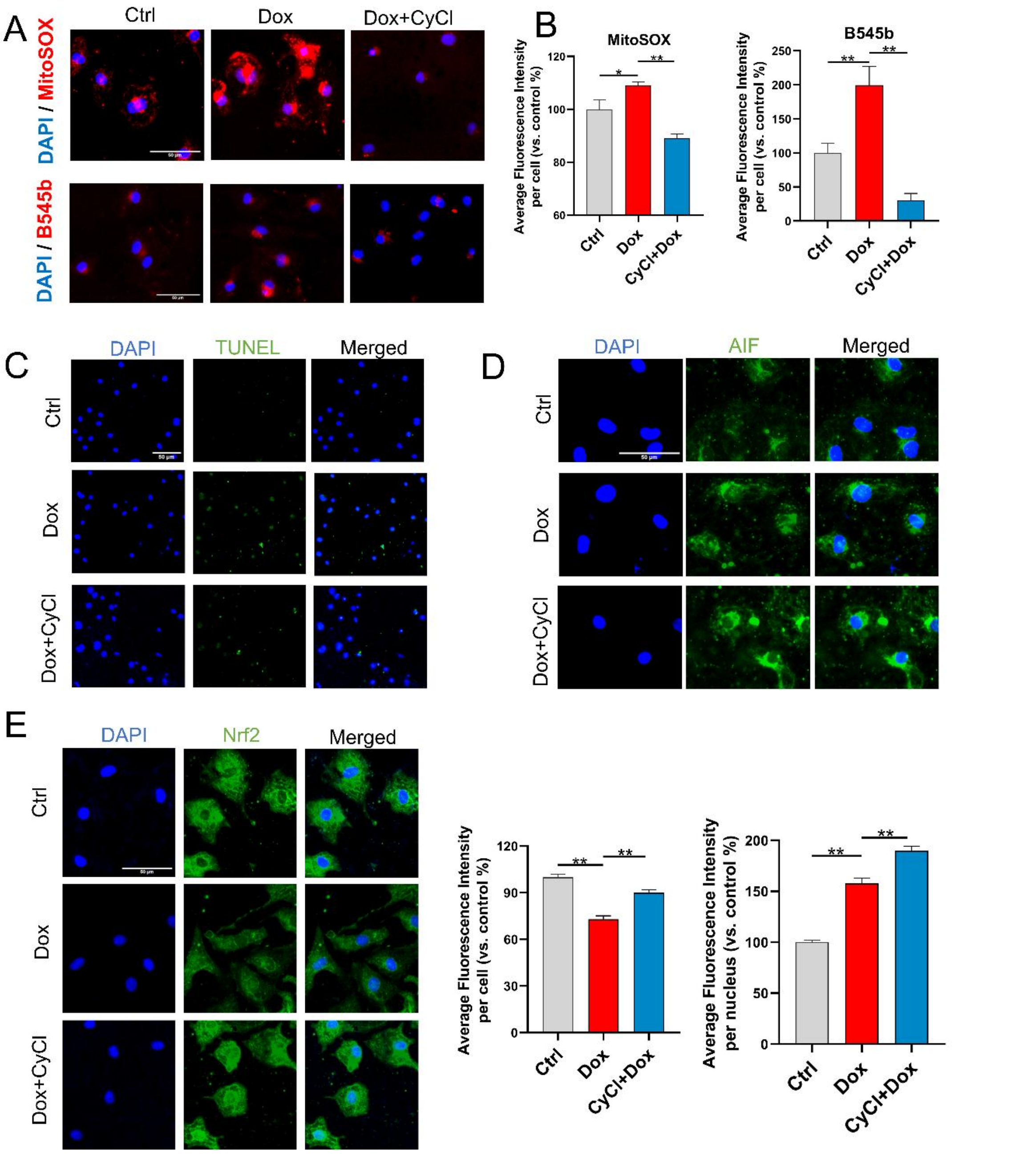

Supporting Information Figure S4. CyCl rescued Dox-induced cardiomyocytes injury in human induced pluripotent stem cell-derived cardiomyocytes (hiPSC-CMs). (A-B) Representative fluorescence images and corresponding quantification of MitoSox and B545b in hiPSC-CMs. (C-D) Representative images of TUNEL staining and AIF immunofluorescence in hiPSC-CMs. (E) Representative fluorescence images and corresponding quantification of Nrf2 immunofluorescence. Scale bar:50μm. Quantitative data are presented as the mean ± SEM. Statistical significance was analyzed using one-way ANOVA with Tukey’s post hoc test; *P < 0.05, **P < 0.01.

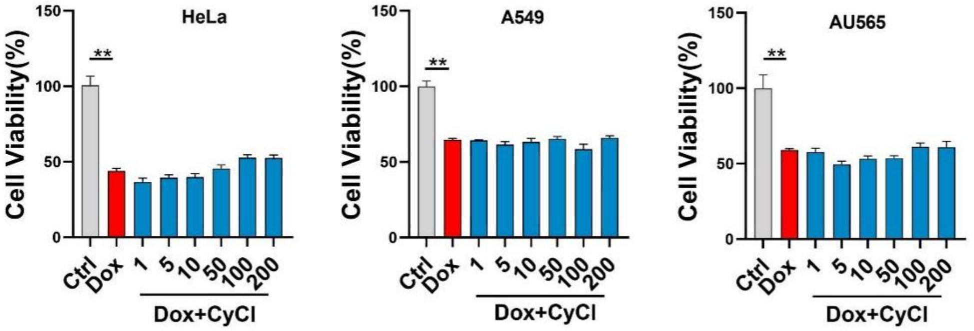

Supporting Information S5. Effects of CyCl on the cell viability of tumor lines HeLa, A549 and AU565. Quantitative data are presented as the mean ± SEM. Statistical significance was analyzed using a one-way ANOVA with Tukey's post hoc test; **P < 0.01.

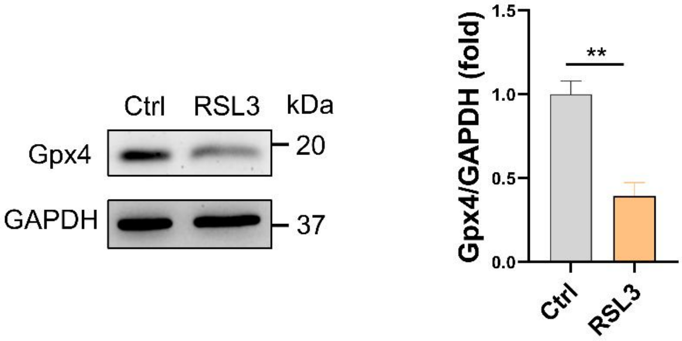

Supporting Information Figure S6. RSL3 inhibits the expression of GPX4 protein level in H9C2 cells. Quantitative data are presented as the mean ± SEM. Statistical significance was analyzed using student t test; **P < 0.01.

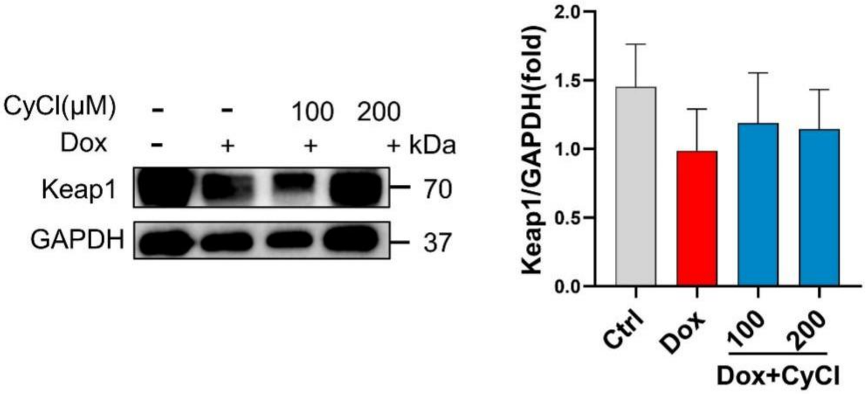

Supporting Information S7. Western blot showing Keap1 protein expression in H9C2 cells (n = 5 per group). Quantitative data are presented as the mean ± SEM. Statistical significance was analyzed using a one-way ANOVA with Tukey's post hoc test.

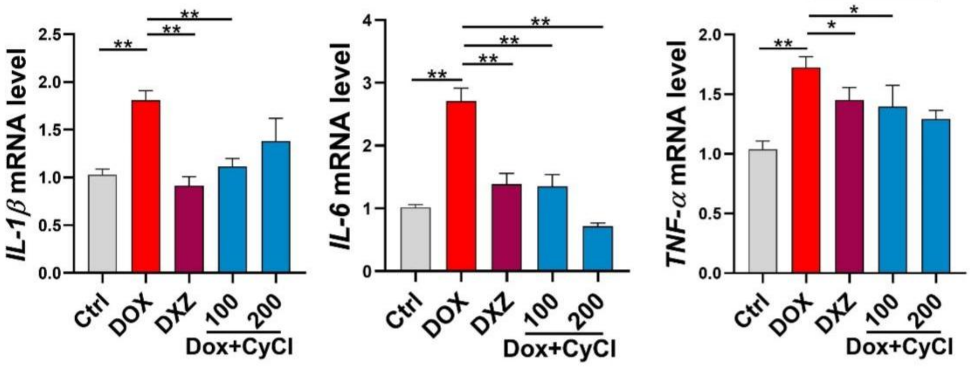

Supporting Information S8. Transcriptional expression of inflammatory cytokines in the acute DIC mice model. Quantitative data are presented as the mean ± SEM. Statistical significance was analyzed using one-way ANOVA with Tukey's post hoc test; *P < 0.05, **P < 0.01.

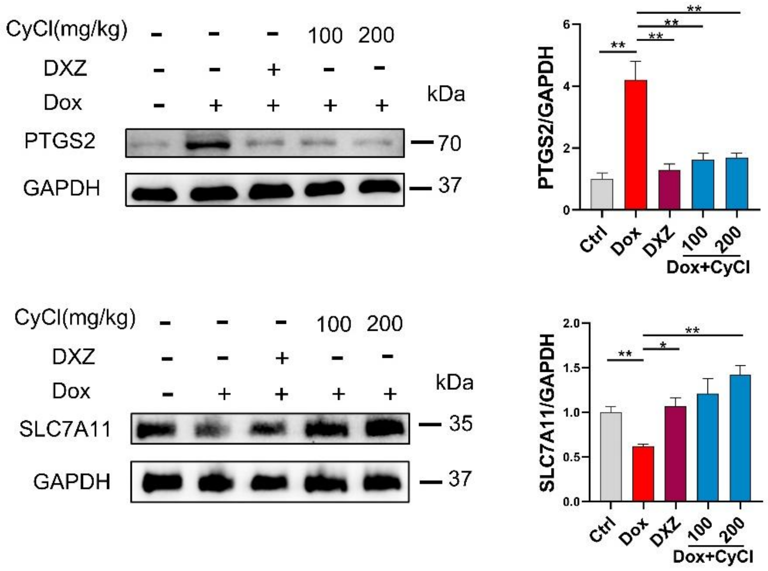

Supporting Information S9. Protein expression of PTGS2 and SLC7A11 in the myocardium tissue of acute DIC mice model with different treatments. Quantitative data are presented as the mean ± SEM. Statistical significance was analyzed using one-way ANOVA with Tukey's post hoc test; *P < 0.05, **P < 0.01.

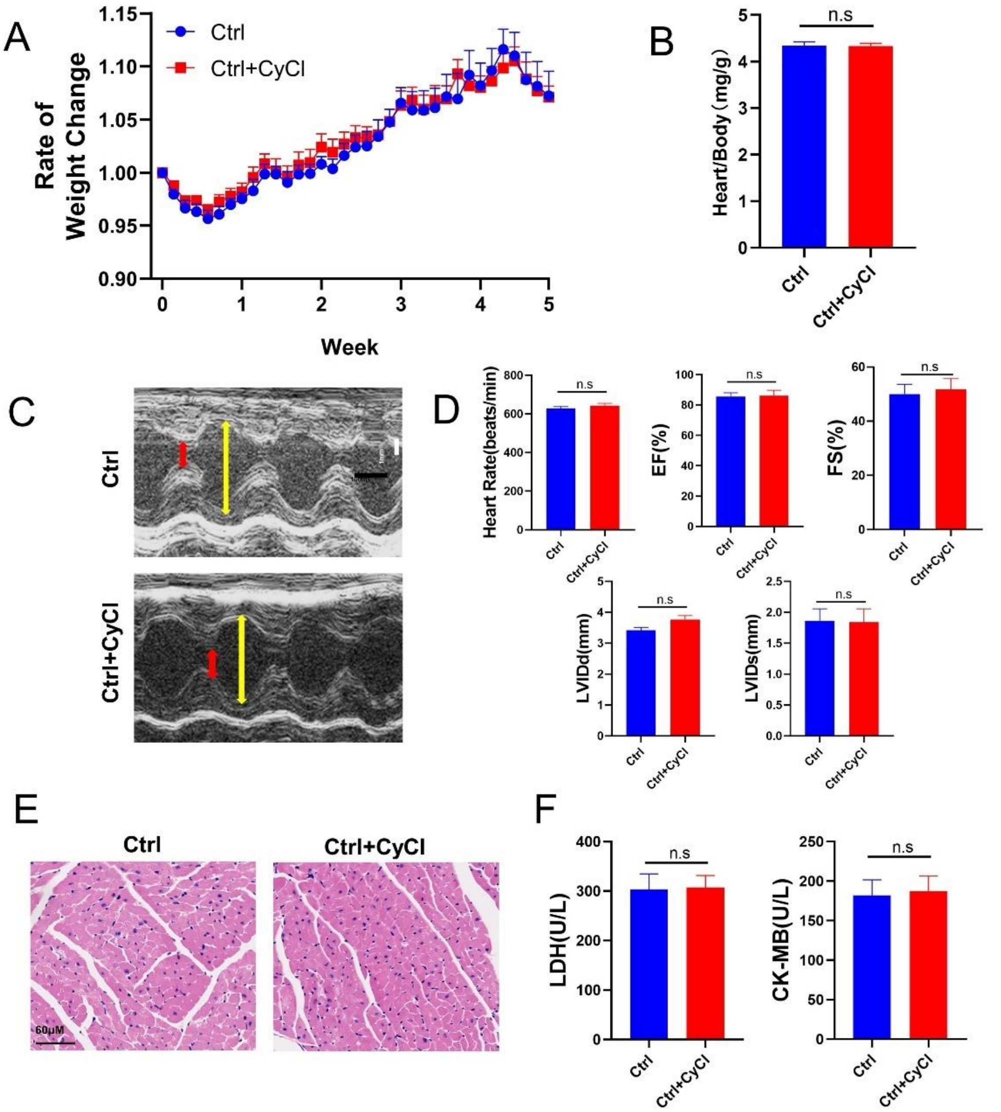

Supporting Information Figure S10. No systemic or cardiac toxicity was observed in mice with long-term CyCl supplementation. (A-B) Body weight and heart weight/body weight ratio of control and CyCl-treated mice (n=7 per group). (C-D) Representative images and quantitative analysis of echocardiogram (n=7 per group). (E) H&E staining of mice cardiac tissue sections. Scar bar,60μm. (F) Serum levels of LDH and CK-MB (n = 7 per group). Quantitative data are presented as the mean ± SEM. Statistical significance was analyzed using Student's t test. ns, non-significant.

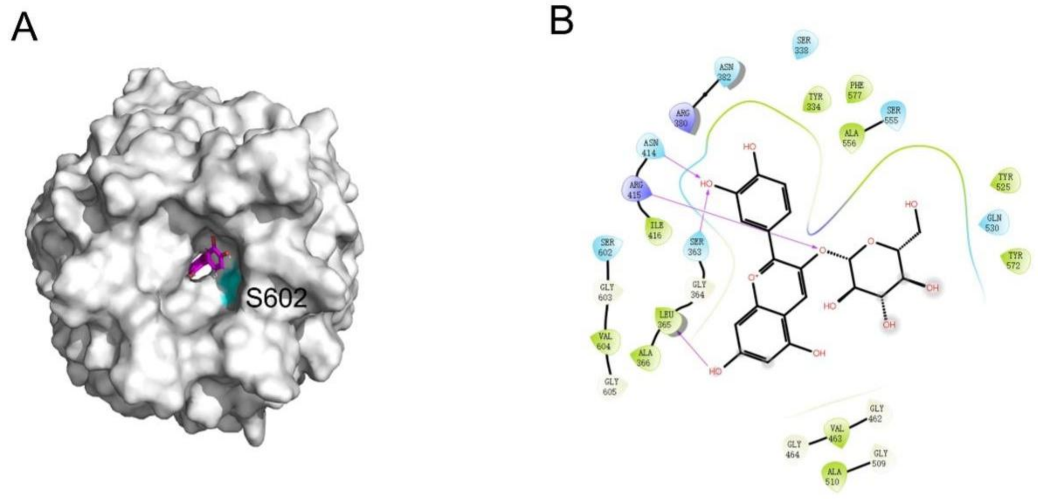

Supporting Information Figure S11. Comparison of molecular docking for binding to Keap1 between CyCl and C3G. (A) The spatial location of CyCl and S602. CyCl and S602 were marked with purple and cyan, respectively. (B)The interaction diagram between C3G and the Kelch1 domain of Keap1. Hydrogen bonds are depicted by the purple arrow.

Supporting Information Table S1: Comparison of ZVSegNet and HRNet with existing work on the validation set.

	IoU	DC	Precision	Recall
**ZVSegNet**	0.88	0.935	0.974	0.902
R2AU-Net	0.852	0.918	0.976	0.871
U-Net	0.847	0.915	0.973	0.868
U-Net 3+	0.849	0.915	0.975	0.869
SAR-U-Net	0.849	0.916	0.975	0.868
Attention-UNet	0.859	0.922	0.975	0.878
R2U-Net	0.871	0.929	0.975	0.891

	RMSE	MAE	R	SD
**HRNet**	18.899	11.859	0.883	13.533
Linear	26.623	16.295	0.76	21.054
FC	26.914	16.943	0.725	20.912
PPnet	25.859	17.472	0.788	19.064

Supporting Information Table S2: Phenotypic screening results.

Rank	Compounds	CAS	Efficacy score	Molecular weight	Chemical Structure Types	Biological activity
1	Rosmarinic acid	20283-92-5	1.1246	360.3	Phenylpropanoids	Anti-inflammatory; antioxidant; antitumor; [1,2] antibacterial [3]
2	Cyanidin Chloride	528-58-5	0.9836	322.7	Flavonoids	Antioxidant;[4] antitumor;[5] antibacterial [6]
3	Mangiferin	4773-96-0	0.8888	422.3	Flavonoids	Antitumor; Antidiabetic;[7] Anti-obesity;[8] Antiulcer; Anti-inflammatory [9]
4	2''-O-Galloylhyperin	53209-27-1	0.8773	616.5	Flavonoids	Anti-inflammatory;[10] antioxidant; Anti-immunity [11]
5	Ginsenoside Re	52286-59-6	0.861	947.2	Terpenes	Antidiabetic;[12,13] Anti-myocardial ischemia-reperfusion injury [14]
6	Lithospermic acid	28831-65-4	0.8509	538.5	Phenylpropanoids	Antioxidant;[15] Neuroprotection [16]
7	Glycyrrhizic acid	85441-51-6	0.8459	888.3	Terpenes	anti-virotic;[17] Anti-inflammatory;[18,19] antitumor
8	Epicatechin	863-03-6	0.8407	442.4	Flavonoids	Anti-hypertension;[20] Anti-inflammatory;[21] antioxidant [22]
9	Chlorogenic acid	327-97-9	0.8345	354.3	Phenylpropanoids	Anti-inflammatory;[23]antioxidant [24]
10	Salidroside	10338-51-9	0.8209	300.3	Phenols	anti-tumor;[25] hpyerglycemic;[26] antioxidant;[27] Neuroprotection [28]
11	Vaccarin	53452-16-7	0.8296	726.6	Flavonoids	antioxidant; [29] Anti-fibrosis;[30] Angiogenesis promotion [31]
12	Protocatechuic Acid	99-50-3	0.8004	154.1	Phenols	Antioxidant; Anti-inflammatory;[31,32] Neuroprotection; antibacterial; anti-virotic; anti-ageing [33]
13	Scutellarin	27740-01-8	0.7873	462.4	Flavonoids	Antioxidant; Anti-inflammatory; anticoagulation [34,35]
14	Monopotassium glycyrrhizinate	911217-00-0	0.7843	894	Terpenes	Anti-inflammatory; anti anaphylaxis
15	Salvianolic acid D	142998-47-8	0.7832	418.3	Phenylpropanoids	Anti-inflammatory; Anti-atherosclerosis [36]
16	Huperzine A	120786-18-7	0.7796	242.32	Alkaloids	Anti-Alzheimer[37–39]
17	Rhodionin	85571-15-9	0.7724	448.377	Flavonoids	Antioxidant; antitumor; Myocardial protection [40]

18	Dimethyl lithospermate B	875313-64-7	0.7599	746.7	Phenylpropanoids	Anti-arrhythmia;[41] antioxidant; Antidiabetic [42]
19	Calceolarioside B	105471-98-5	0.7505	478.4	Phenylpropanoids	Antithrombosis [43]
20	Herbacetin	527-95-7	0.7402	302.2	Flavonoids	Antioxidant; anti-virotic; Anti-inflammatory; antitumor;[44] Antidiabetic [45,46]
21	β-Carotene	7235-40-7	0.7248	536.9	Terpenes	Anti-ASD;[47] antitumor;[48] antioxidant [49]
22	Ginsenoside Rg2	52286-74-5	0.7238	785	Terpenes	Myocardial protection;[50] antitumor;[51] Neuroprotection [52]
23	Catechin	154-23-4	0.7108	290.3	Flavonoids	Antioxidant;[53] antibacterial [54,55]

Supporting Information Table S3: Sequences of the primers used for real-time RT-PCR analysis

Gene name	Species	Forward primer(5'→3')	Reverse primer(5'→3')
EF-1α	Zebrafish	AGAAGGCTGCCAAGACCAAG	AGAGGTTGGGAAGAACACGC
Ptgs2	Zebrafish	TGGATCTTTCCTGGGTGAAGG	GAAGCTCAGGGGTAGTGCAG
GAPDH	Rat	CCGCATCTTCTTGTGCAGTG	GAGAAGGCAGCCCTGGTAAC
Ptgs2	Rat	ATGTTCGCATTCTTTGCCCAG	TACACCTCTCCACCGATGAC
Fth1	Rat	CCCTTTGCAACTTCGTCGCT	CTCCGAGTCCTGGTGGTAGT
Slc7a11	Rat	TACCTGCAGGGCAATGTGAG	TGAAGATGCCCGATCCGATG
β-actin	Mouse	GTGACGTTGACATCCGTAAAGA	GCCGGACTCATCGTACTCC
GAPDH	Mouse	ATCATCCCTGCATCCACT	ATCCACGACGGACACATT
Ptgs2	Mouse	CTGCGCCTTTTCAAGGATGG	GGGGATACACCTCTCCACCA
Fth1	Mouse	TCCTGGCTTGGGTTGATTGG	CGGCAAATCATCTCCTCCACT
Slc7a11	Mouse	TGGCGGTGACCTTCTCTGA	ACAAAGATCGGGACTGCTAATGA
Hamp1	Mouse	GCACCACCTATCTCCATCAACA	TTCTTCCCCGTGCAAAGG
IL-1β	Mouse	CCCAACTGGTACATCAGCAC	TCTGCTCATTCACGAAAAGG
IL-6	Mouse	ACAACCACGGCCTTCCCTACTT	CACGATTTCCCAGAGAACATGTG
TNF-α	Mouse	CATGAGCACAGAAAGCATGATCCG	AGCAGGAATGAGAAGAGGCTGAG

Supporting Information Video S1: Heartbeat video of control Tg(cmyb:eGFP) zebrafish.

Supporting Information Video S2: Heartbeat video of Dox-treated Tg(cmyb:eGFP) zebrafish.

Supporting Information Video S3: Blood flow video of control Tg(LCR:eGFP) zebrafish.

Supporting Information Video S4: Blood flow video of Dox-treated Tg(LCR:eGFP) zebrafish.

References

[1] C. H. Chung, W. Jung, H. Keum, T. W. Kim, S. Jon, *ACS Nano* **2020**, *14*, 6887.

[2] J. Li, Q. Duan, X. Wei, J. Wu, Q. Yang, *Small* **2022**, e2204388.

[3] "Rosmarinic acid inhibits Ca2+-dependent pathways of T-cell antigen receptor-mediated signaling by inhibiting the PLC-gamma 1 and Itk activity - PubMed," can be found under https://pubmed.ncbi.nlm.nih.gov/12511421/, **n.d.**

[4] Z. Cheng, X. Si, H. Tan, Z. Zang, J. Tian, C. Shu, X. Sun, Z. Li, Q. Jiang, X. Meng, Y. Chen, B. Li, Y. Wang, *Crit Rev Food Sci Nutr* **2021**, 1.

[5] C. Liu, L. Zhu, K. Fukuda, S. Ouyang, X. Chen, C. Wang, C.-J. Zhang, B. Martin, C. Gu, L. Qin, S. Rachakonda, M. Aronica, J. Qin, X. Li, *Sci Signal* **2017**, *10*, eaaf8823.

[6] X. Su, H. Yu, X. Wang, C. Zhang, H. Wang, X. Kong, Y. Qu, Y. Luan, Y. Meng, J. Guan, G. Song, L. Wang, W. Song, Y. Zhao, *Virulence* **2022**, *13*, 1434.

[7] M. Wang, Z. Zhang, Q. Huo, M. Wang, Y. Sun, H. Liu, J. Chang, B. He, Y. Liang, *ACS Appl Mater Interfaces* **2022**, *14*, 11092.

[8] M. S. Rahman, Y.-S. Kim, *Metabolism* **2020**, *107*, 154228.

[9] M. Mahmoud-Awny, A. S. Attia, M. F. Abd-Ellah, H. S. El-Abhar, *PLoS One* **2015**, *10*, e0132497.

[10] S.-D. Zhang, P. Wang, J. Zhang, W. Wang, L.-P. Yao, C.-B. Gu, T. Efferth, Y.-J. Fu, *Chem Biol Interact* **2019**, *304*, 20.

[11] P. Wang, C. Gao, N. Guo, S.-D. Zhang, W. Wang, L.-P. Yao, J. Zhang, T. Efferth, Y.-J. Fu, *Front Pharmacol* **2018**, *9*, 679.

[12] J.-T. Xie, S. R. Mehendale, X. Li, R. Quigg, X. Wang, C.-Z. Wang, J. A. Wu, H. H. Aung, P. A Rue, G. I. Bell, C.-S. Yuan, *Biochim Biophys Acta* **2005**, *1740*, 319.

[13] A. S. Attele, Y.-P. Zhou, J.-T. Xie, J. A. Wu, L. Zhang, L. Dey, W. Pugh, P. A. Rue, K. S. Polonsky, C.-S. Yuan, *Diabetes* **2002**, *51*, 1851.

[14] C.-X. Bai, K. Takahashi, H. Masumiya, T. Sawanobori, T. Furukawa, *Br J Pharmacol* **2004**, *142*, 567.

[15] "Lithospermic acid attenuates 1-methyl-4-phenylpyridine-induced neurotoxicity by blocking neuronal apoptotic and neuroinflammatory pathways - PubMed," can be found under https://pubmed.ncbi.nlm.nih.gov/26018660/, **n.d.**

[16] "Rosmarinic acid antagonized 1-methyl-4-phenylpyridinium (MPP+)-induced neurotoxicity in MES23.5 dopaminergic cells - PubMed," can be found under https://pubmed.ncbi.nlm.nih.gov/20966113/, **n.d.**

[17] T. Tong, H. Hu, J. Zhou, S. Deng, X. Zhang, W. Tang, L. Fang, S. Xiao, J. Liang, *Small* **2020**, *16*, e1906206.

[18] C. Bailly, G. Vergoten, *Pharmacol Ther* **2020**, *214*, 107618.

[19] M. Choi, L. T. Thuy, Y. Lee, C. Piao, J. S. Choi, M. Lee, *ACS Appl Mater Interfaces* **2021**, *13*, 47313.

[20] I. Bernatova, *Biotechnol Adv* **2018**, *36*, 666.

[21] K. Kawai, N. H. Tsuno, J. Kitayama, Y. Okaji, K. Yazawa, M. Asakage, S. Sasaki, T. Watanabe, K. Takahashi, H. Nagawa, *J Allergy Clin Immunol* **2005**, *115*, 186.

[22] B. S. Kim, J. Leong, S. J. Yu, Y. Cho, C. G. Park, D.-H. Kim, E. Ko, S. G. Im, J. Lee, Y. J. Kim, H. Kong, *Small* **2019**, *15*, e1900765.

[23] S. Mansoori, A. Dini, S. C. Chai, *Ageing Res Rev* **2021**, *66*, 101254.

[24] "Apple polyphenol extracts prevent damage to human gastric epithelial cells in vitro and to rat gastric mucosa in vivo - PubMed," can be found under https://pubmed.ncbi.nlm.nih.gov/15647180/, **n.d.**

[25] L. Yang, Y. Yu, Q. Zhang, X. Li, C. Zhang, T. Mao, S. Liu, Z. Tian, *Artif Cells Nanomed Biotechnol* **2019**, *47*, 3500.
[26] L. Ju, X. Wen, C. Wang, Y. Wei, Y. Peng, Y. Ding, L. Feng, L. Shu, *Front Pharmacol* **2017**, *8*, 749.
[27] Y.-F. Wang, Y.-Y. Chang, X.-M. Zhang, M.-T. Gao, Q.-L. Zhang, X. Li, L. Zhang, W.-F. Yao, *Phytomedicine* **2022**, *99*, 154020.
[28] Z. Xie, H. Lu, S. Yang, Y. Zeng, W. Li, L. Wang, G. Luo, F. Fang, T. Zeng, W. Cheng, *Front Pharmacol* **2020**, *11*, 568423.
[29] L. T, Y. X, Z. X, W. Y, Z. M, C. W, H. B, X. F, Q. L, *Frontiers in pharmacology* **2022**, *13*, DOI 10.3389/fphar.2022.956247.
[30] "Vaccarin alleviates hypertension and nephropathy in renovascular hypertensive rats - PubMed," can be found under https://pubmed.ncbi.nlm.nih.gov/29399101/, **n.d.**
[31] F. Xie, L. Feng, W. Cai, Y. Qiu, Y. Liu, Y. Li, B. Du, L. Qiu, *Mol Med Rep* **2015**, *12*, 1131.
[32] M. H. Yang, S. H. Baek, A. Chinnathambi, S. A. Alharbi, K. S. Ahn, *Phytother Res* **2021**, *35*, 1953.
[33] A. Me, S. Gsg, S. Gm, S. Ha, *Environmental toxicology and pharmacology* **2021**, *83*, DOI 10.1016/j.etap.2020.103577.
[34] J. Song, Y. He, C. Luo, B. Feng, F. Ran, H. Xu, Z. Ci, R. Xu, L. Han, D. Zhang, *Pharmacol Res* **2020**, *161*, 105109.
[35] L. Wang, Q. Ma, *Pharmacol Ther* **2018**, *190*, 105.
[36] J. Xi, Y. Rong, Z. Zhao, Y. Huang, P. Wang, H. Luan, Y. Xing, S. Li, J. Liao, Y. Dai, J. Liang, F. Wu, *J Ethnopharmacol* **2021**, *271*, 113855.
[37] W. Zhang, J. Song, W. Li, D. Kong, Y. Liang, X. Zhao, G. Du, *Mediators Inflamm* **2020**, *2020*, 9049614.
[38] N. Ghassab-Abdollahi, K. Mobasseri, A. Dehghani Ahmadabad, H. Nadrian, M. Mirghafourvand, *Phytother Res* **2021**, *35*, 4971.
[39] J. Yue, B. R. Dong, X. Lin, M. Yang, H. M. Wu, T. Wu, *Cochrane Database Syst Rev* **2012**, *12*, CD008827.
[40] P. Khanal, F. Zargari, B. F. Far, D. Kumar, M. R, Y. K. Mahdi, N. K. Jubair, S. K. Saraf, P. Bansal, R. Singh, M. Selvaraja, Y. N. Dey, *Front Pharmacol* **2021**, *12*, 785964.
[41] Y. Chen, M. Tang, S. Yuan, S. Fu, Y. Li, Y. Li, Q. Wang, Y. Cao, L. Liu, Q. Zhang, *Oxid Med Cell Longev* **2022**, *2022*, 1348795.
[42] J. M. Fish, D. R. Welchons, Y.-S. Kim, S.-H. Lee, W.-K. Ho, C. Antzelevitch, *Circulation* **2006**, *113*, 1393.
[43] E. Lim, J. Ricci, M. Jung, *Molecules* **2011**, *16*, 9886.
[44] X. Zheng, P. Pu, B. Ding, W. Bo, D. Qin, G. Liang, *Food Chem* **2021**, *362*, 130237.
[45] D. J. Kim, E. Roh, M.-H. Lee, N. Oi, D. Y. Lim, M. O. Kim, Y.-Y. Cho, A. Pugliese, J.-H. Shim, H. Chen, E. J. Cho, J.-E. Kim, S. C. Kang, S. Paul, H. E. Kang, J. W. Jung, S.-Y. Lee, S.-H. Kim, K. Reddy, Y. I. Yeom, A. M. Bode, Z. Dong, *Cancer Res* **2016**, *76*, 1146.
[46] X. Wei, Z. Zhao, R. Zhong, X. Tan, *J Ethnopharmacol* **2021**, *279*, 114356.
[47] "Herbacetin, a flaxseed flavonoid, ameliorates high percent dietary fat induced insulin resistance and lipid accumulation through the regulation of hepatic lipid metabolizing and lipid-regulating enzymes - PubMed," can be found under https://pubmed.ncbi.nlm.nih.gov/29653099/, **n.d.**
[48] Y. Avraham, D. Mankuta, L. Lipsker, L. Vorobiev, S. Patael, G. Hassid, E. M. Berry, A. Albeck, *Bioorg Chem* **2021**, *115*, 105224.
[49] S. Kacar, E. Sariisik, V. Sahinturk, *Naunyn Schmiedebergs Arch Pharmacol* **2022**, *395*, 407.
[50] A. Nishino, H. Yasui, T. Maoka, *J Oleo Sci* **2017**, *66*, 77.
[51] G. Liu, J. Zhang, F. Sun, J. Ma, X. Qi, *Biomed Res Int* **2022**, *2022*, 8866660.

[52] H. Jeon, Y. Jin, C.-S. Myung, K.-S. Heo, *Arch Pharm Res* **2021**, *44*, 702.
[53] J. Cui, R. Shan, Y. Cao, Y. Zhou, C. Liu, Y. Fan, *J Ethnopharmacol* **2021**, *266*, 113466.
[54] C. Musial, A. Kuban-Jankowska, M. Gorska-Ponikowska, *Int J Mol Sci* **2020**, *21*, E1744.
[55] J. V. Higdon, B. Frei, *Crit Rev Food Sci Nutr* **2003**, *43*, 89.
[56] W. C. Reygaert, *Biomed Res Int* **2018**, *2018*, 9105261.
